# Establishing a Role for Bacterial Cellulose in Environmental Interactions: Lessons Learned from Diverse Biofilm-Producing *Proteobacteria*

**DOI:** 10.3389/fmicb.2015.01282

**Published:** 2015-11-17

**Authors:** Richard V. Augimeri, Andrew J. Varley, Janice L. Strap

**Affiliations:** Molecular Microbial Biochemistry Laboratory, Faculty of Science, University of Ontario Institute of TechnologyOshawa, ON, Canada

**Keywords:** plant–bacteria interactions, animal–bacteria interactions, fungal–bacteria interactions, ecophysiology, bacterial cellulose, biofilms, c-di-GMP, *Komagataeibacter (Gluconacetobacter) xylinus*

## Abstract

Bacterial cellulose (BC) serves as a molecular glue to facilitate intra- and inter-domain interactions in nature. Biosynthesis of BC-containing biofilms occurs in a variety of *Proteobacteria* that inhabit diverse ecological niches. The enzymatic and regulatory systems responsible for the polymerization, exportation, and regulation of BC are equally as diverse. Though the magnitude and environmental consequences of BC production are species-specific, the common role of BC-containing biofilms is to establish close contact with a preferred host to facilitate efficient host–bacteria interactions. Universally, BC aids in attachment, adherence, and subsequent colonization of a substrate. Bi-directional interactions influence host physiology, bacterial physiology, and regulation of BC biosynthesis, primarily through modulation of intracellular bis-(3′→5′)-cyclic diguanylate (c-di-GMP) levels. Depending on the circumstance, BC producers exhibit a pathogenic or symbiotic relationship with plant, animal, or fungal hosts. *Rhizobiaceae* species colonize plant roots, *Pseudomonadaceae* inhabit the phyllosphere, *Acetobacteriaceae* associate with sugar-loving insects and inhabit the carposphere, *Enterobacteriaceae* use fresh produce as vehicles to infect animal hosts, and *Vibrionaceae*, particularly *Aliivibrio fischeri*, colonize the light organ of squid. This review will highlight the diversity of the biosynthesis and regulation of BC in nature by discussing various examples of *Proteobacteria* that use BC-containing biofilms to facilitate host–bacteria interactions. Through discussion of current data we will establish new directions for the elucidation of BC biosynthesis, its regulation and its ecophysiological roles.

## Introduction

A characteristic common to all domains of life is the ubiquitous presence of cellulose. It is found in vascular plants, green algae, oomycetes, hyphochytriomycetes, tunicates, and numerous bacterial species (Römling, 2002; Stone, 2005; Kimura and Itoh, 2007; Sarkar et al., 2009). Plant cellulose (PC) is the most abundant source of cellulose. As a major constituent of cotton and wood, cellulose is of immense economic value.

**FIGURE 1:**
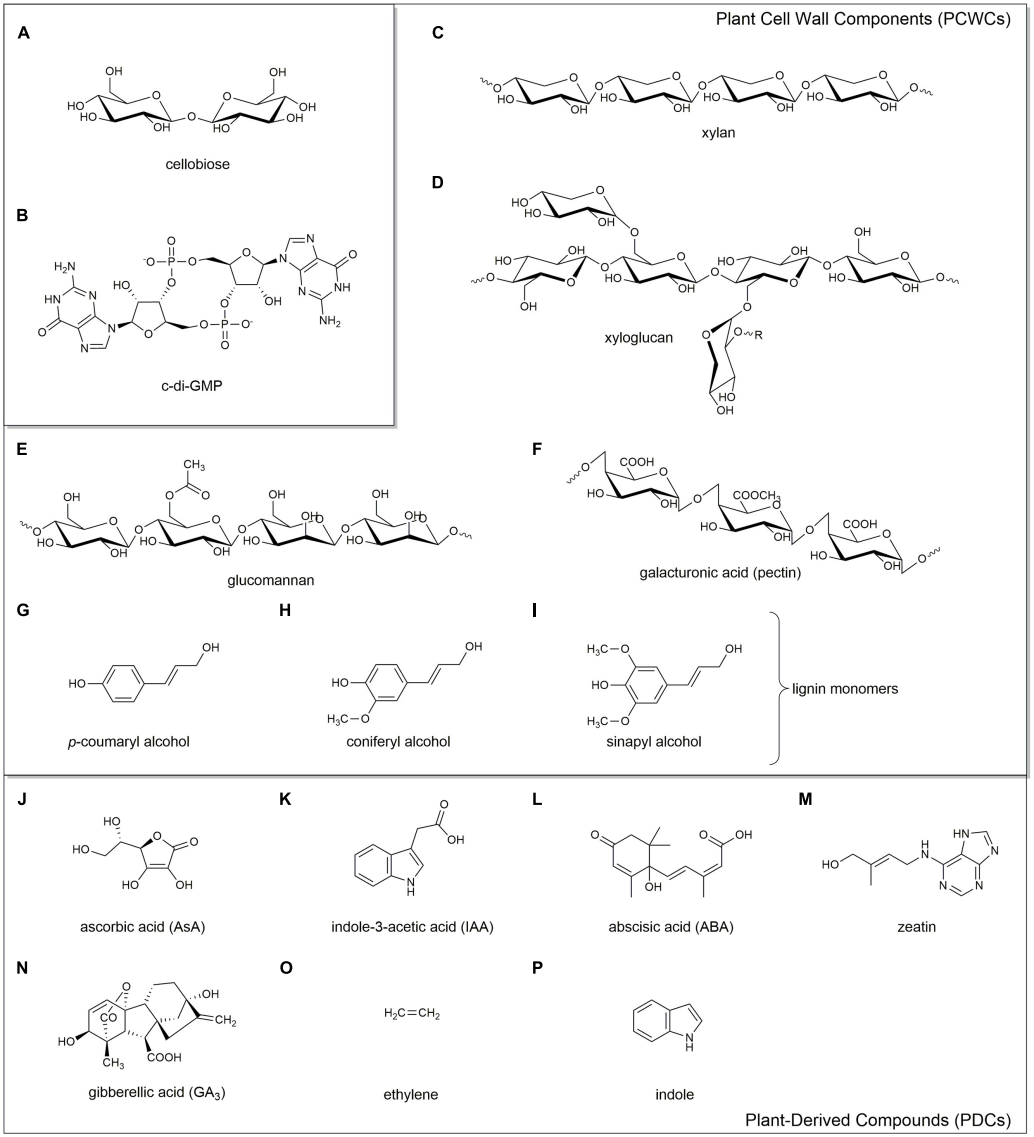
**Chemical structures of the key compounds discussed in this review.** Cellobiose **(A)**, a proposed monomeric unit of cellulose; c-di-GMP **(B)**, the key secondary messenger involved in biofilm production. Plant cell wall components (PCWCs): hemicelluloses xylan **(C)**, xyloglucan **(D)**, and glucomannan **(E)**; pectin **(F)**; and lignin monomers *p*-coumaryl alcohol **(G)**, coniferyl alcohol **(H)**, and sinapyl alcohol **(I)**. Plant-derived compounds (PDCs): ascorbic acid **(J)**, indole-3-acetic acid **(K)**, abscisic acid **(L)**, zeatin **(M)**, gibberellic acid **(N)**, ethylene **(O)**, and indole **(P)**.

Structurally, cellulose is an unbranched biopolymer consisting of β-D-glucopyranose units that are connected through β-1,4-glycosidic linkages. Each of the β-D-glucopyranose residues are rotated 180° with respect to its neighbor, making cellobiose (**Figure 1A**) the monomeric unit of cellulose (O’Sullivan, 1997). The two dominant allomorphs of cellulose, cellulose I and cellulose II, differ in their stability, crystallinity, and H-bonding patterns (Kroon-Batenburg and Kroon, 1997). Cellulose I is less stable and more crystalline due to the highly ordered H-bonding patterns of its parallel glucan chains. In contrast, cellulose II is more stable, but less crystalline due to a less ordered H-bonding pattern (Yu and Atalla, 1996; Somerville, 2006).

Cellulose is the main constituent in the cell wall of vascular plants where it is in complex with hemicelluloses, pectins, the aromatic polymer lignin and numerous glycoproteins (Heredia et al., 1995; Somerville, 2006). Synthesis of PC is achieved through the action of cellulose synthase complexes, consisting of cellulose synthase (CESA) proteins arranged into rosettes. PC synthesis and CESA proteins have been thoroughly reviewed elsewhere (Richmond and Somerville, 2000; Mutwil et al., 2008; Carpita, 2011; Carroll and Specht, 2011; Endler and Persson, 2011; Kumar and Turner, 2014; Li et al., 2014). It should be noted that PC synthase complexes have a bacterial origin, as the encoding genes were obtained through cyanobacterial endosymbiosis (Nobles et al., 2001; Nobles and Brown, 2004).

**FIGURE 2:**
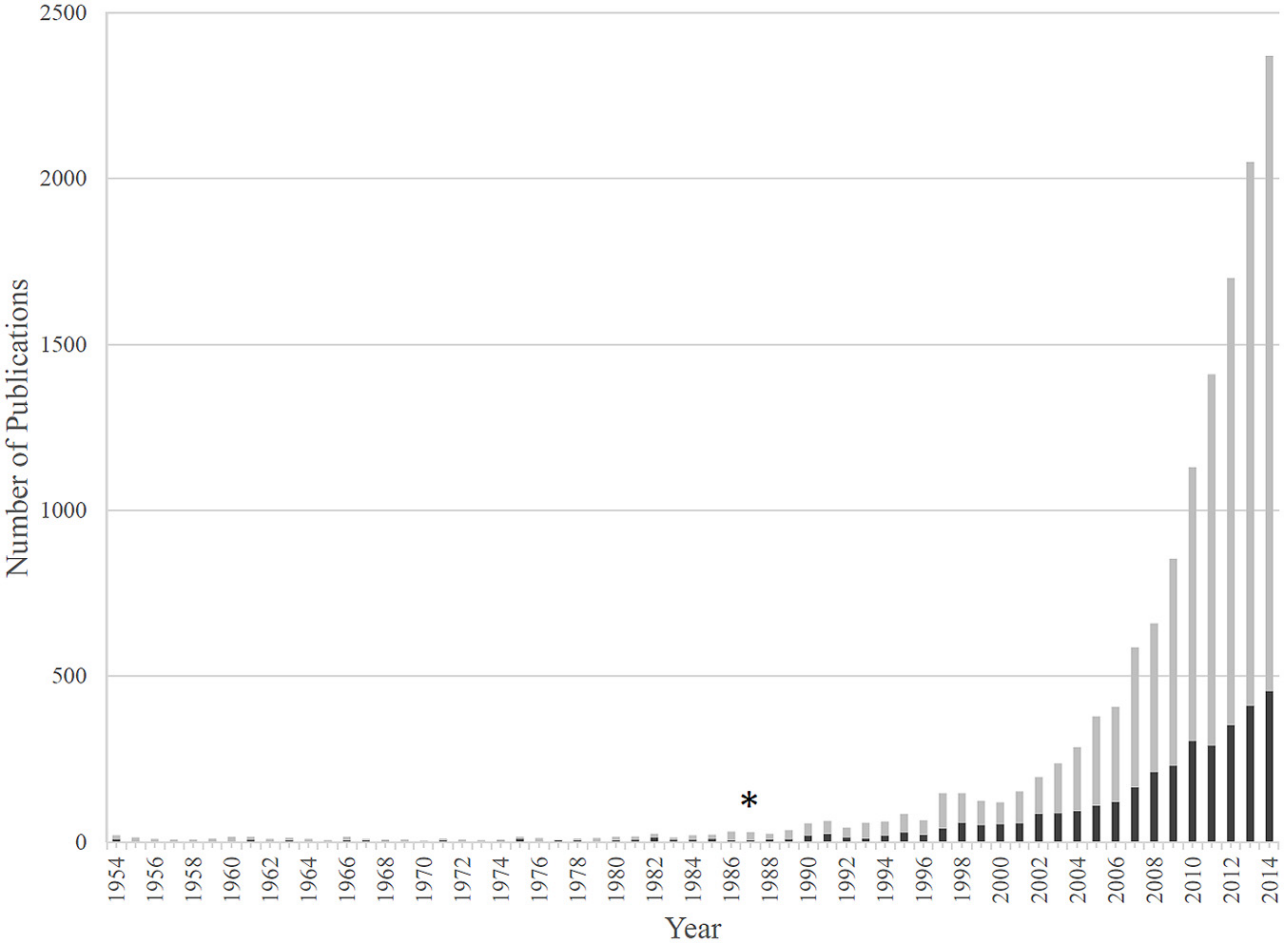
**The number of publications regarding BC and *Komagataeibacter xylinus*.** BC publications are shown by gray bars, while those studying BC production by *K. xylinus* are shown by black bars. The 1987 discovery that c-di-GMP activates BcsA and controls BC production marks a turning point for research in the bacterial cellulose (BC) field (^∗^).

In addition to plants, cellulose synthesis has been observed in numerous microorganisms, such as green algae and oomycetes, which use cellulose in their cell walls (Richmond, 1991; Fugelstad et al., 2009), as well as various bacterial species (Römling and Galperin, 2015). Genera of bacterial cellulose (BC) producers include *Komagataeibacter* (formerly *Gluconacetobacter*), *Gluconacetobacter* (formerly *Acetobacter*), *Enterobacter*, *Pseudomonas, Achromobacter, Alcaligenes, Aerobacter, Azotobacter, Agrobacterium, Burkholderia, Dickeya, Escherichia, Rhizobium, Salmonella*, and *Sarcina* (Ross et al., 1991; Römling, 2002; Römling and Galperin, 2015). The most prominent phylum of BC producers are the *Proteobacteria*, which inhabit diverse ecological niches (**Table 1**). Research regarding BC synthesis spans seven decades (Aschner and Hestrin, 1946; Hestrin et al., 1947). Interest in BC synthesis in the model organism, *Komagataeibacter xylinus*, has increased steadily in the last 15 years (**Figure 2**) due to the improvement of next-generation sequencing technologies, the publication of the genome sequences of numerous BC producers, and the increased availability of genetic tools (Römling and Galperin, 2015).

**Table 1:** Diversity of experimentally proven BC producers in the phylum *Proteobacteria*.

Class	Order	Family	Genus	Host	Reference^1^
α*-proteobacteria*	*Rhizobiales*	*Rhizobiaceae*	*Agrobacterium*	Plant	Matthysse et al., 1981
			*Rhizobium*	Plant	Laus et al., 2005
		*Acetobacteriaceae*	*Komagataeibacter*	Plant	Brown et al., 1976
			*Asaia*	Animal	Kumagai et al., 2011
β*-proteobacteria*	*Neisseriales*	*Chromobacteriaceae*	*Chromobacterium*	Animal	Recouvreux et al., 2008
γ*-proteobacteria*	*Enterobacteriales*	*Enterobacteriaceae*	*Enterobacter*	Plant/animal	Hungund and Gupta, 2010
			*Escherichia*	Plant/animal	Ellermann et al., 2015
			*Salmonella*	Plant/animal	Römling et al., 2004
			*Dickeya*	Plant	Jahn et al., 2011
	*Pseudomonadales*	*Pseudomonadaceae*	*Pseudomonas*	Plant/animal	Spiers et al., 2003
	*Vibrionales*	*Vibrionaceae*	*Aliivibrio*	Animal	Bassis and Visick, 2010

*^1^Select reports demonstrating BC production from the respective bacterial genus.*

Despite decades of study, there is much to learn regarding the environmental interactions mediated by BC. Bacteria synthesize cellulose using BC synthesis (Bcs) proteins encoded by the *bcs* operon. Four *bcs* genes (*bcsABCD*) were initially identified and characterized in the acetic acid bacterium *K. xylinus* (Saxena et al., 1990, 1994; Wong et al., 1990). Since then, genome sequences of numerous bacterial strains were shown to contain organizationally diverse *bcs* operons. The molecular biology of the various *bcs* operons and their encoded proteins have been reviewed (Römling, 2002; Römling and Galperin, 2015). Briefly, BcsA is an integral inner membrane protein with transmembrane (TM) domains in clusters of 4+4 (Omadjela et al., 2013; Kumar and Turner, 2014). It contains a very small N-terminal domain and a large intracellular catalytic glycosyltransfersase domain. The C-terminus consists of a bis-(3′→5′)-cyclic diguanylate- (cyclic di-GMP or c-di-GMP; **Figure 1B**) binding PilZ domain, which controls the activity of the catalytic domain through conformational changes (Amikam and Galperin, 2006; Ryjenkov et al., 2006; Römling et al., 2013; Morgan et al., 2014). BcsB is a periplasmic protein attached to BcsA by a single C-terminal TM helix and contains two carbohydrate binding domains (CBD1 and CBD2) that chaperone the synthesized glucan chain through the periplasm (Morgan et al., 2013). The functional BcsA subunit is stabilized by BcsB (Morgan et al., 2013). BcsA and BcsB are the only two proteins required for *in vitro* cellulose synthesis, though *in vitro*-formed BC is less crystalline (Wong et al., 1990; Omadjela et al., 2013). BcsC and BcsD are required for maximal BC production and crystallization *in vivo* (Wong et al., 1990; Saxena et al., 1994). BcsC, an outer membrane pore (Whitney et al., 2011) and BcsD, a periplasmic protein (Hu et al., 2010), couple the export and crystallization of BC microfibrils. Römling and Galperin (2015) proposed a model for the organization of the entire BC synthase complex based on crystal structure data of the *Rhodobacter sphaeroides* BcsA-BcsB complex, the BcsC-like AlgK-AlgE protein complex of *Pseudomonas aeruginosa*, and the BcsD protein of *K. xylinus*. In addition to the *bcs* operon, numerous ancillary genes are involved in the regulation, synthesis, crystallization and export of BC; these have been reviewed elsewhere (Ross et al., 1991; Römling, 2002; Römling and Galperin, 2015).

Since the glucan chains must be exported through the peptidoglycan layer in the Gram-negative cell wall, perturbations of the peptidoglycan network affect the export and the crystallization of the BC microfibril (see Deng et al., 2015 for schematic). *Komagataeibacter hansenii* ATCC 23769 mutants defective in genes encoding for lysine decarboxylase and alanine racemase produce less crystalline BC than wild type (Deng et al., 2015) suggesting that a highly structured peptidoglycan network is required for proper ribbon assembly.

Structurally, BC is more pure than PC in that it lacks hemicellulose (**Figures 1C–E**), pectin (**Figure 1F**), and lignin (**Figures 1G–I**). BC from *K. xylinus* exhibits a higher crystallinity index and degree of polymerization than PC (Gayathry and Gopalaswamy, 2014; Kumar and Turner, 2014). This may be explained by the presence of the BcsD protein that is unique to the BC synthase complex (Delmer, 1999) and shown to be involved in crystallization (Saxena et al., 1994; Sunagawa et al., 2013). These structural characteristics, along with the ability to form BC-nanocomposites have made BC of great interest to numerous industries, particularly those involved in drug-delivery systems, medical devices, food products and acoustics; these have been reviewed elsewhere (Iguchi et al., 2000; Siró and Plackett, 2010; Nwodo et al., 2012; Abeer et al., 2014).

Some BC producers make soluble hemicellulose-like extracellular polysaccharides (EPSs) that contain glucose, mannose, rhamnose, galactose, and glucuronic acid in variable molar ratios (Fang and Catchmark, 2014, 2015). Most of the EPS can be removed by solvent precipitation of culture supernatant, but some EPS cannot be removed. This “hard to extract” EPS (HE-EPS) complexes with the BC matrix (Fang and Catchmark, 2014, 2015). By binding in between adjacent glucan chains, HE-EPS impact cellulose ribbon assembly and crystallization (Fang and Catchmark, 2014; Deng et al., 2015) by disrupting the highly ordered H-bonding pattern of crystalline BC resulting in an amorphous matrix. This is analogous to the incorporation of hemicellulosic-polysaccharides into the cellulose matrix of plant cell walls.

Though the structures of PC and BC are similar, their functions are different. PC is a structural component of the plant cell wall and is essential to plant survival. In contrast, BC is not essential for survival but does confer a survival advantage. When BC producers are grown statically in liquid, they either form a solid surface-associated biofilm (SSAB) at the bottom of the vessel, or build a floating biofilm at the air–liquid interface (ALI), commonly referred to as a pellicle. Biofilms are multicellular, surface-associated microbial communities embedded within an extracellular matrix comprised of polysaccharides, proteins, and extracellular DNA (eDNA; Geesey et al., 1978; Costerton et al., 1995; Flemming and Wingender, 2010). For aerobic bacteria, the ALI is a favorable environment for BC production as it provides high concentrations of oxygen from the air, while still allowing access to nutrients present in the soluble medium. Pellicle formation has been thoroughly studied in regards to the Gram-positive bacterium *Bacillus subtilis* (Vlamakis et al., 2013), but is less well-characterized in Gram-negative bacteria. The current knowledge of pellicle formation in Gram-negative bacteria has been recently reviewed (Armitano et al., 2014).

**FIGURE 3:**
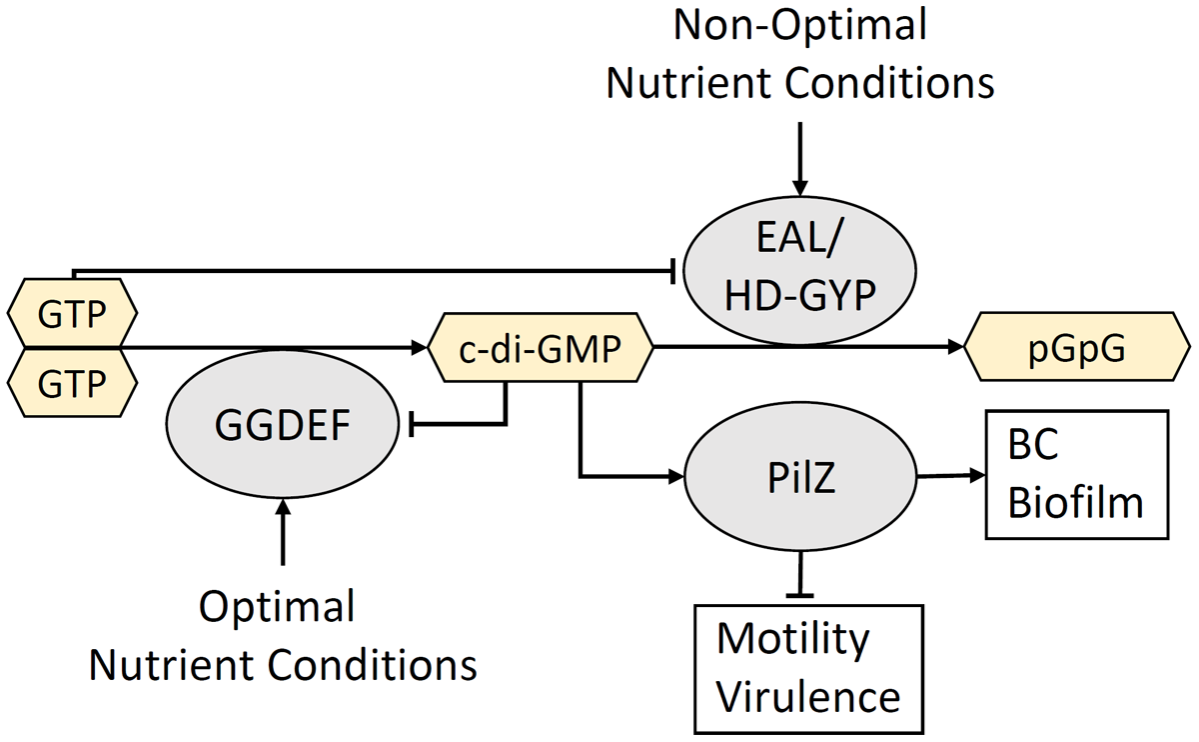
**The turnover of c-di-GMP is controlled by environmental conditions.** Extracellular cues that signal conditions suitable for colonization activate GGDEF domain-containing DGC enzymes that synthesize c-di-GMP. High levels of c-di-GMP binds the PilZ domain within the BcsA glycosyltransferase and triggers the production of a BC-containing biofilm. In contrast, extracellular signals associated with an unsuitable growth environment activate EAL/HD-GYP domain-containing PDEs that degrade c-di-GMP and produce the linear dinucleotide, pGpG. The resulting low c-di-GMP and high pGpG levels activate motility and virulence mechanisms that allow the bacterium to move to a more optimal environment for colonization. Negative feedback occurs between c-di-GMP and DGCs, as well as between GTP and PDEs.

In the environment, SSABs have numerous implications for host–bacteria interactions. Bacteria that form SSABs can switch between a sessile, biofilm-forming state and a motile, planktonic state depending on environmental signals (Flemming and Wingender, 2010). This adaptive mechanism allows motile bacterial species to search for a suitable growth environment. Once a nutrient-rich substrate is found, growth and biofilm production commence to initiate colonization. This transition is controlled by the antagonistic action of diguanylate cyclases (DGCs) and phosphodiesterases (PDEs) that contain conserved GGDEF and EAL or HD-GYP domains, respectively (Galperin et al., 2001; Simm et al., 2004). These catalytic domains are responsible for the synthesis (GGDEF) and degradation (EAL and HD-GYP) of the ubiquitous bacterial second messenger and activator of the BC synthase, c-di-GMP (Ross et al., 1987; Amikam and Benziman, 1989). Bi-functional GGDEF-EAL and GGDEF-HD-GYP enzymes also exist. Many of these proteins contain upstream sensory domains that control the activity of the downstream catalytic domains in response to environmental cues. These sensory domains come in different flavors, such as the Per-Arnt-Sim (PAS) and GAF domains, the latter being named after the proteins in which it is found: cGMP-specific PDEs, adenylyl cyclases and FhlA (*Escherichia coli*). These sensory domains contain prosthetic groups, such as heme, flavin mononucleotide, flavin adenine dinucleotide, and various chromophores which allow proteins to sense a variety of signals including O_2_ (Chang et al., 2001; Gilles-Gonzalez and Gonzalez, 2004), the redox status of the cell (Qi et al., 2009) and light (Tarutina et al., 2006). Binding of these ligands modulates the activity of the catalytic GGDEF, EAL, and HD-GYP domains and couples environmental signals with the turnover of c-di-GMP. Mechanisms involved in c-di-GMP signaling have previously been reviewed by Hengge (2009) and Römling et al. (2013). Typically, low-levels of c-di-GMP is a cellular signal for motility and virulence, while high levels of c-di-GMP initiates the transition to a biofilm-forming state by activating enzymes involved in biofilm synthesis (**Figure 3**).

The remainder of this review will focus on defining key bacteria that produce BC biofilms in the environment, particularly symbionts and pathogens that interact with plants and animals (**Table 2**). We will highlight the role of BC biofilms in host colonization, identify various environmental interactions that occur between bacteria and their hosts and discuss the regulation of BC biosynthesis from different environmental perspectives. The ecological diversity of BC production, and its involvement in inter-domain interactions will be highlighted.

**Table 2:** Bacterial cellulose (BC) producers and host–bacteria interactions discussed in detail in this review.

Bacterium	Host	Location	Relationship	Reference^1^
*Rhizobium leguminosarum*	Plant	Roots	Mutualistic	Lin et al., 2015
*Agrobacterium tumefaciens*	Plant	Roots	Pathogenic	Tolba and Soliman, 2014
*Komagataeibacter xylinus*	Plant	Fruit	Uncertain	Neera et al., 2015
*Escherichia coli*	Human	Gut	Pathogenic	Manageiro et al., 2015
	Plant	Fresh produce	Uncertain	Rangel-Vargas et al., 2015
*Salmonella enterica*	Human	Gut	Pathogenic	Manageiro et al., 2015
	Plant	Fresh produce	Uncertain	Rangel-Vargas et al., 2015
*Asaia* sp.	Insect	Gut	Symbiotic	Favia et al., 2007
*Aliivibrio fischeri*	Squid	Light organ	Symbiotic	Boettcher and Ruby, 1990

*^1^References given are, to our knowledge, the most recent reports describing the isolation of the respective bacterium from its respective host.*

## Plant–Bacteria Interactions of BC Producers

### Root–bacteria Interactions

The term rhizosphere was first coined in 1904 by the German botanist Lorenz Hiltner to describe the area surrounding plant roots (Hiltner, 1904). The nutrient-rich rhizosphere is a complex microenvironment harboring an array of microorganisms that interact with plant roots and influence plant physiology, including symbiotic and pathogenic species. BC production by rhizosphere bacteria enhances their ability to attach to and establish close contact with root hairs. Two well-studied BC producing *Rhizobiaceae*: *Agrobacterium tumefaciens*, a tumor-inducing phytopathogen, and *Rhizobium leguminosarum*, a nitrogen-fixing plant symbiont will be discussed to highlight the role BC plays in these root–bacteria interactions.

#### *Rhizobium* sp.

**FIGURE 4:**
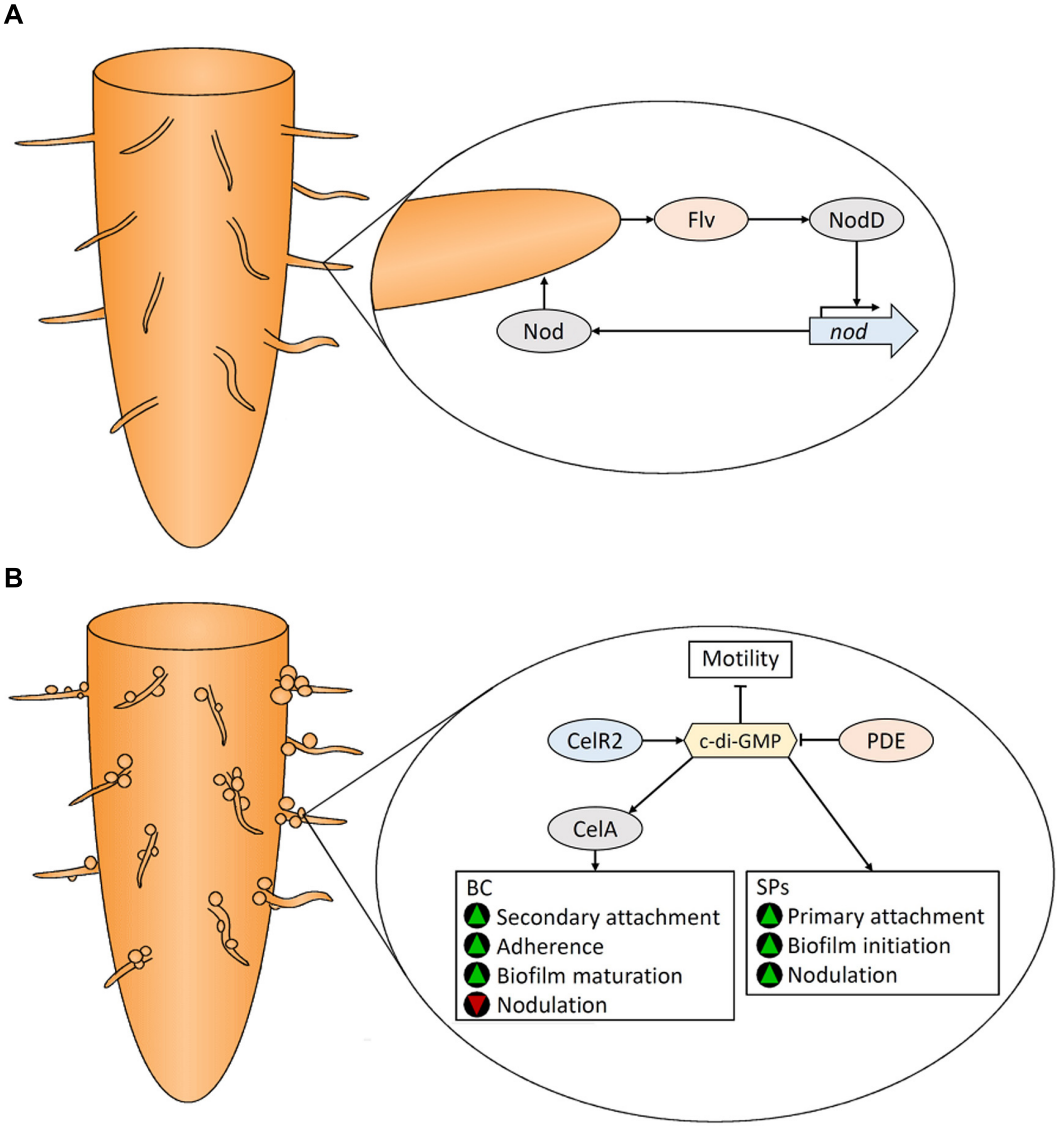
**Colonization of legume roots by *Rhizobium* spp. Plant-derived flavonoids (Flv) and Rhizobium Nod factors positively regulate each other (A).** Bacterial cellulose (BC) and surface polysaccharides (SPs) are produced by Rhizobium to assist in attachment and subsequent biofilm formation on plant roots **(B)**. See text for details. Green triangles indicate that a characteristic would be promoted, while red triangles indicate that a characteristic would be reduced.

Within the rhizosphere, certain *Rhizobium* species participate in endosymbiotic relationships with their legume (*Fabaceae*) hosts. These bacteria produce nodules on roots where they fix atmospheric nitrogen (N_2_) into ammonia using the nitrogenase enzyme. In return, the bacteria are nourished by plant-produced nutrients. Due to the agricultural importance of rhizobia–legume mutualism, this interaction has been well-studied. Nodulation (*nod*) genes, primarily located on large symbiotic plasmids (pSyms; Brom et al., 1992; Zhang et al., 2001) encode Nod factors, a family of lipo-chito-oligosaccharide signaling compounds that facilitate the infection process. Transcription of *nod* genes is induced by flavonoids (Cooper, 2004) and other compounds that are secreted with root exudate (see review: Janczarek et al., 2015). Nitrogen-limiting conditions cause legume roots to secrete flavonoid compounds (Graham, 1991) that elicit a chemotactic response (Munoz Aguilar et al., 1988) attracting rhizobia to root hairs where colonization occurs (Fournier et al., 2008). Flavonoids are detected by the constitutively expressed bacterial NodD protein which positively regulates other *nod* genes (Spaink et al., 1987). A positive feedback loop is established as plant-derived flavonoids, which themselves are induced by Nod factors, stimulate Nod factor production in the bacterium (**Figure 4A**). Curling of the root hair tip entraps attached bacterial cells (Esseling et al., 2003), which through the action of Nod factors, induces cell wall modification by plant enzymes (van Spronsen et al., 1994) and through production of cell wall degrading cellulases (Robledo et al., 2012) and pectate lyases (Xie et al., 2012; Downie and Xie, 2015). Plant cell wall degradation facilitates production of an infection thread allowing entry of bacteria into the plant cell. Nodulation commences when rhizobia-produced Nod factors and phytohormones (see review: Ferguson and Mathesius, 2014) bind to specific plant receptors initiating a cascade of morphogenic processes in the root (Jones et al., 2007).

##### Surface polysaccharides synthesized by Rhizobium sp.

In addition to Nod factors, the production of surface polysaccharides (SPs) are involved in establishing bacteroids during rhizobia–legume symbiosis (**Figure 4B**). Plant cells contain receptors that are induced by Nod factors that subsquently perceive bacterial SPs (Kawaharada et al., 2015). Root hair attachment by rhizobia occurs in two stages (Dazzo et al., 1984). Primary attachment to root surfaces is faciliated by the unipolar polysaccharide (UPP), a glucomannan SP that binds root hair lectins (Laus et al., 2006). UPP is produced from one end of the cell for polar attachment. Its essential role in colonization is illustrated by the fact that UPP-deficient mutants cannot bind to root hairs in conditions representive of soil (Laus et al., 2006). In addition, rhizobia produce an array of strain-specific EPS that assist in adherence and initiation of symbiosis. Over-production of EPS increases biofilm formation and nodulation (Fujishige et al., 2006). EPS non-producing mutants are unable to infect root hairs, underscoring the influence of EPS on root colonzation. Bacterial lipopolysaccharides (LPSs) and capsular polysaccharides (K-antigens or KPS) also play a significant role (Becker et al., 2005). Symbiosis requires LPS containing the O-antigen in addition to KPS; mutants with LPS lacking this substituent and KPS-deficient mutants were both unable to infect hosts or fix nitrogen (Putnoky et al., 1988; Fraysse et al., 2003). Rhizobia also secrete cyclic-β-glucans into the periplasmic space which play a role in root colonization (Bhagwat et al., 1999). For details regarding these SPs, and other protein factors involved in root hair attachment see Janczarek et al. (2015).

Bacterial cellulose is also produced by rhizobia (Napoli et al., 1975) where it is involved in the secondary stages of rhizobial attachment to plant roots (Smit et al., 1987) and biofilm maturation (**Figure 4B**); cells that have reached the secondary attachment stage demonstrate improved adherence (Dazzo et al., 1984; Smit et al., 1992; Ausmees et al., 1999). BC, however, is not required for nodulation (Smit et al., 1987; Ausmees et al., 1999). Laus et al. (2005) showed that BC production inhibits nodulation by causing agglutination of bacterial cells.

##### Regulation of BC biosynthesis in Rhizobium sp.

*Rhizobium* BC biosynthesis is controlled by two operons, *celABCE* and *celR1*-*celR2* (Ausmees et al., 1999). CelA encodes a BC synthase that is activated by c-di-GMP (Ausmees et al., 2001). There are two isozymes of CelC: CelC1 is encoded on the pSym plasmid, while CelC2 (also known as BcsZ; see review: Römling and Galperin, 2015) is encoded in the *celABCE* operon (Jimenéz-Zurdo et al., 1996). CelC2, a cellulase that degrades PC in the cell wall of root hairs, is essential for symbiotic infection of legume roots (Robledo et al., 2008). Wild type levels of CelC2 are essential to produce BC fibrils of the optimal length for biofilm formation and root colonization (Robledo et al., 2012). Disruption and over-expression of the the DGC gene, *celR2*, abolished and enhanced BC production, respectively (Ausmees et al., 2001) indicating that CelR2 produces c-di-GMP that activates CelA (**Figure 4B**). The PDE responsible for c-di-GMP degradation has not been identified in rhizobia.

High levels of intracellular c-di-GMP reduces motility and enhances BC biosynthesis and biofilm formation in *R. leguminosarum* (Pérez-Mendoza et al., 2014), culminating in improved attachment to roots. It is unknown how BC gene expression is regulated in rhizobia, but plant compounds have been implicated (Ausmees et al., 1999; Laus et al., 2005). c-di-GMP hampers the later stages of rhizobia-legume symbiosis, but promotes a plant-associated, versus a saprophytic lifestyle since *Rhizobium* mutant strains with elevated c-di-GMP levels exhibit reduced plant growth promoting abilities and lower numbers of bacterial cells within root nodules (Pérez-Mendoza et al., 2014). Details regarding the metabolism of c-di-GMP during legume root colonization remain to be elucidated.

#### Agrobacterium tumefaciens

Infection by *A. tumefaciens* is facilitated by transfer of the oncogenic T-DNA fragment, from its tumor-inducing (Ti) plasmid into the nuclear genome of a broad range of dicotyledonous plants. T-DNA encodes enzymes responsible for the biosynthesis of plant growth-promoting auxin and cytokinin phytohormones resulting in tumors characteristic of crown gall disease. The process of and plant-response to *A. tumefaciens* infection has been reviewed elsewhere (Pitzschke, 2013), as have the bacterial responses to plant-derived signaling molecules (Subramoni et al., 2014).

##### Roles of EPS in A. tumefaciens attachment to plant roots

*Agrobacterium tumefaciens* utilizes various EPS to anchor itself to plant roots. Similar to rhizobia, biofilm production and irreversible polar attachment of *A. tumefaciens* to roots requires UPP (Tomlinson and Fuqua, 2009). Enzymes responsible for UPP biosynthesis by *A. tumefaciens* are encoded by the genes *atu1235*, *atu1236*, *atu1237*, *gumB* (*atu1238*), and *exoP* (*atu1239*; Matthysse, 2014). UPP is produced when *A. tumefaciens* comes into contact with a surface (Tomlinson and Fuqua, 2009; Li et al., 2012; Xu et al., 2013) and is absent in planktonic cells (Li et al., 2012). Currently it is unknown how surface-induced UPP production is activated.

*Agrobacterium tumefaciens* produces BC that coalesces with PC on plant surfaces (Matthysse et al., 1981). BC biosynthesis in *A. tumefaciens* requires enzymes encoded by the *celABCG* and *celDE* operons (Matthysse et al., 1995) that have been described in detail elsewhere (Römling and Galperin, 2015). Over-production of BC increases biofilm formation on roots and enhances colonization ability but does not restore virulence in an avirulent *A. tumefaciens* strain (Matthysse et al., 2005). Root attachment can occur in the absence of UPP and BC, but it is extremely weak and involves less bacterial cells (Sykes and Matthysse, 1986) due to the action of other adherence factors such as pili (Wang et al., 2014), adhesins (Mongiardini et al., 2008), and other EPS.

Biosynthesis of other EPS occurs in *A. tumefaciens.* Mutants that cannot produce cyclic-β-1,2-D-glucan in the periplasm are defective in plant cell attachment (Douglas et al., 1982), but since exogenous addition does not improve attachment (Puvanesarajah et al., 1985), a role for periplasmic EPS in the process is suggested. Biosynthesis of curdlan, a linear β-1,3-glucan, occurs primarily when cell growth is repressed under conditions of limited nitrogen and abundant carbon source (McIntosh et al., 2005). Under these conditions, *A. tumefaciens* embeds into the curdlan matrix and binds to roots where nutrients are available. Deletion of the curdlan synthase gene has no effect on biofilm formation and attachment (Xu et al., 2012). The most abundant EPS produced by *A. tumefaciens* under laboratory conditions is acidic succinoglycan (Hisamatsu et al., 1978). *A. tumefaciens* mutants unable to produce succinoglycan retain the ability to form biofilms and attach to plant surfaces (Tomlinson et al., 2010). Similar to *Rhizobium* species, succinoglycan may assist in *A. tumefaciens* attachment to roots, although there is no direct evidence for this. Based on the available data, the roles of these EPS in biofilm formation and attachment to plant surfaces remains obscure.

##### Regulation of biofilm polysaccharide biosynthesis

**FIGURE 5:**
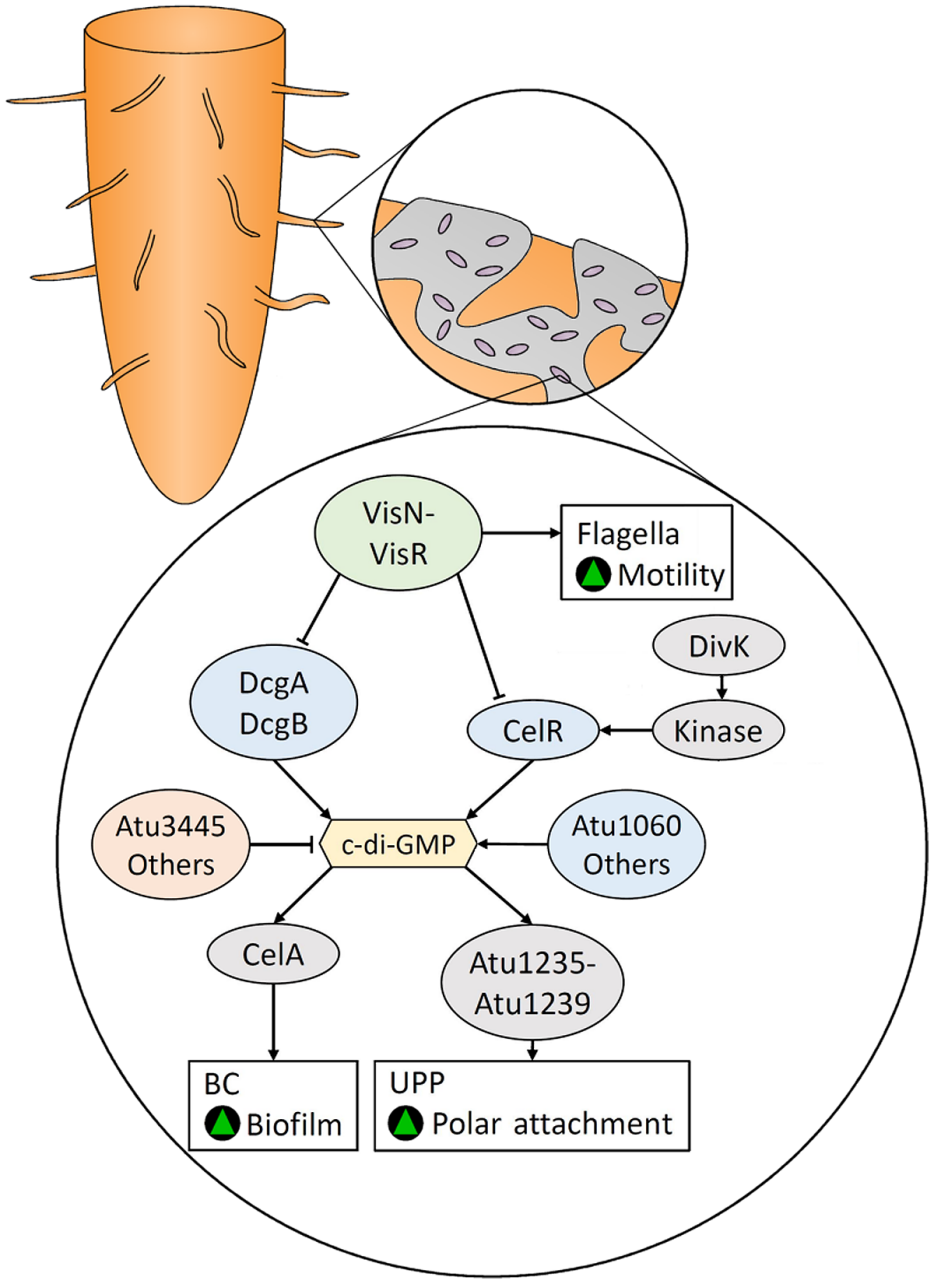
**Bacterial cellulose- and unipolar polysaccharide (UPP)-mediated root colonization in *Agrobacterium tumefaciens* is regulated by c-di-GMP, which is controlled by various DGCs and PDEs.** The positive regulators of flagellar-mediated motility, VisN and VisR, inhibit the activity of DGCs and are responsible for the inverse regulation of motility and biofilm formation. Green triangles indicate that a characteristic would be promoted.

Bacterial cellulose and UPP are co-regulated by c-di-GMP in *A. tumefaciens* (**Figure 5**). The stimulatory effect of c-di-GMP on BC biosynthesis in *A. tumefaciens* was first reported by Amikam and Benziman (1989). The genome of *A. tumefaciens* encodes 33 enzymes that are predicted to be DGCs and/or PDEs (Heindl et al., 2014) underscoring their importance to the physiology of this bacterium. Xu et al. (2013) demonstrated that elevated levels of c-di-GMP increases UPP and BC production, while VisN and VisR, activators of flagellar motility (Sourjik et al., 2000), act to inhibit UPP and BC biosynthesis by repressing the DGCs DcgA, DcgB, and CelR (**Figure 5**). They also showed that mutations in the PDE, Atu3495, results in increased levels of c-di-GMP, UPP and BC while reducing motility. Additionally, over-expression of the DGC genes, *celR* and *atu1060*, increases BC production, while deletion of *celR*, but not *atu1060*, decreases BC production (Michael Barnhart et al., 2013).

Enhancement of BC biosynthesis by CelR is dependent on DivK (Barnhart et al., 2014), a regulator of cell division and putative response regulator (**Figure 5**). DivK is encoded by the *divK*-*celR* operon, which is homologous to the *celR1*–*celR2* operon in rhizobia. Disruption of the *divK* gene results in CelR being unable to stimulate BC biosynthesis, although constitutive expression of CelR restored the wild type phenotype. DivK acts upstream of the activation of CelR, by interacting with other proteins in a DivK-CelR signaling pathway (Barnhart et al., 2014). Both DivK and CelR contain a pair of CheY-like domains that can be phosphorylated, suggesting the DivK-CelR pathway involves kinases.

Feirer et al. (2015) demonstrated that the bi-functional GGDEF-EAL domain protein, DcpA, is modulated by a pteridine reductase (PruA) responsible for the biosynthesis of a novel pterin compound. In the presence of this compound, the EAL domain of DcpA is bound and activated. In its absence, DcpA acts as a DGC. PruR, which contains a predicted molybdopterin-binding domain, was implicated to act as an intermediate between PruA and DcpA through a yet to be identified mechanism. Therefore, the pterin compound produced by PruA has a negative role in attachment and biofilm formation by decreasing levels of c-di-GMP, UPP, and BC.

Another bi-functional GGDEF-EAL domain protein, AvHaCE (encoded by the *avi_3097* gene), has been identified in *Agrobacterium vitis* S4 (Nesbitt et al., 2015). AvHaCE contains both DGC and PDE activity, but does not contain the RxxD motif characteristic of the c-di-GMP binding inhibitory allosteric I-site. Disruption of the EAL domain resulted in the enzyme producing large quantities of c-di-GMP *in vitro*, but its role *in vivo* was not assessed. In addition, disruption of *celG*, located within the *cel* cluster, and *celI*, located elsewhere in the genome, results in over-production of BC (Matthysse et al., 2005). CelI belongs to the MarR/ArsR family of transcriptional regulators, suggesting that CelI acts to down-regulate BC biosynthesis at a transcriptional level.

Bacterial cellulose and UPP play a key role in biofilm formation and the attachment of *A. tumefaciens* to plant roots. The biosynthesis of these polysaccharides is regulated by the intracellular c-di-GMP levels that are modulated by various DGCs and PDEs. However, other environmental and nutritional factors have functional and regulatory roles in surface attachment by *A. tumefaciens*; reviewed elsewhere (Heindl et al., 2014).

### Fruit–bacteria Interactions of the Model BC Producer, *K. xylinus*

Many BC producers colonize fruit. Similar to the ability of *Rhizobiaceae* to colonize plant roots, BC biofilm production enhances colonization of the fruit substrate by serving as a molecular anchor. Two examples of BC producers that have been isolated from decaying fruit are *Enterobacter amnigenus* GH-1 (Hungund and Gupta, 2010), a human pathogen (Capdevila et al., 1998), and *Komagataeibacter* sp., which have been studied in regards to their interaction with fruit.

*Komagataeibacter* are Gram-negative α*-proteobacteria* that belong to the family *Acetobacteraceae* (Cleenwerck et al., 2009; Mamlouk and Gullo, 2013) and are model organisms for BC synthesis. These bacteria produce a crystalline BC pellicle at the ALI of statically grown liquid cultures (Gromet-Elhanan and Hestrin, 1963) and form biofilms on climacteric (Park et al., 2003; Dellaglio et al., 2005; Jahan et al., 2012) and non-climacteric (Valera et al., 2011; Barata et al., 2012) fruit. *Komagataeibacter* species are also commonly isolated from spoiled batches of wine (Bartowsky and Henschke, 2008). The natural habitat of *K. xylinus* is the carposphere; its occurrence in wine is merely a result of its presence on the grapes used to make the wine. Studying the ecophysiology of *K. xylinus* will provide important clues for how the energetically costly BC is regulated and structurally influenced by its environment.

Nutrient availability on the surface of unripe fruits is limited. The small pool of plant-derived nutrients are from exudates or from wounds on the fruit surface. Bacteria are chemotactic toward exudates. For example, plant growth-promoting *Pseudomonas fluorescens* exhibits chemotaxis toward tomato root exudate, some amino acids, malic acid and citric acid but not sugars (de Weert et al., 2002). *E. coli* is chemotactic toward sugars (Adler et al., 1973).

Since *K. xylinus* is an acetic acid bacterium that colonizes fruit, it likely persists within the gut of insects (see Insect–bacteria Interactions). The fruit fly, *Drosophila*, preferentially deposits bacteria on the wounds of fruit where nutrients are most plentiful (Janisiewicz et al., 1999). The pH of these wounds is around 3.5 (Janisiewicz et al., 1999) which is similar to the pH of *K. xylinus* cultures during exponential growth (Qureshi et al., 2013). Bacterial growth within fruit wounds has been observed for a saprophytic strain of *Pseudomonas syringae* (Janisiewicz and Marchi, 1992), another plant-associated BC producer that resides in the phyllosphere (Arrebola et al., 2015). *K. xylinus* persists on unripe fruit in the environment. As fruit ripens, stored starch and cellulose are enzymatically degraded into glucose providing a substrate for growth and BC synthesis (Ahmed and Labavitch, 1980; Brady, 1987).

Williams and Cannon (1989) investigated the environmental roles of *K. xylinus* BC production and studied the fruit–microbe interactions of this bacterium. When *K. xylinus* was inoculated on apple slices, it colonized the substrate and outcompeted fungi and other bacteria when BC was produced, or over-produced, compared to BC non-producers. BC protected *K. xylinus* from dessication and from the damaging effects of UV radiation. *K. xylinus* produces BC to increase environmental fitness which differs from the notion that BC biosynthesis was required to suspend bacteria to the ALI of liquid cultures; an environment where they are not naturally found. This highlights the importance of studying the fruit–bacteria interactions of *K. xylinus*, to better understand the ecological role of BC.

#### Molecular Biology of Fruit Colonization by *K. xylinus*

The *K. xylinus* fruit–bacteria interactions described by Williams and Cannon (1989) demonstrated that BC production is required for effective colonization of apple slices. Therefore, any disruption in BC biosynthesis could be inferred to negatively impact fruit colonization. In *K. xylinus*, BC is synthesized by BC synthesis gene products (BcsA, BcsB, BcsC, BcsD), a cellulose-complimenting protein (CcpAx), an endoglucanase (CmcAx), and a β-glucosidase (BglAx). The absence of any of these proteins results in decreased BC production (Wong et al., 1990; Nakai et al., 2002, 2013; Deng et al., 2013). Interestingly, CmcAx is homologous to the *R. leguminosarum* endoglucanase, CelC2, which degrades the non-crystalline tip of root hairs to make a localized hole that the bacteria can penetrate (Robledo et al., 2008). Similarily, secreted CmcAx (Koo et al., 1998) may also function to degrade the plant cell wall so that *K. xylinus* can obtain valuble nutrients trapped inside. This mechanism may play a role in the persistence of *K. xylinus* on unripe fruit, where nutrients are scarce. CmcAx is secreted by BC non-producers, suggesting a role for CmcAx that is independent of BC biosynthesis.

In addition to BC synthesis machinery, proteins that control the turnover of c-di-GMP influence BC production post-transcriptionally through the activation of BcsA. Three *cdg* (cyclic diguanylate) operons encode DGCs and PDEs that display hierarchical-control of BC biosynthesis in *K. xylinus* (Tal et al., 1998). Disruption of each DGC resulted in reduced BC production *in vivo* (Tal et al., 1998) suggesting they are required for fruit colonization. The PDE, AxPDEA1, contains a heme-based PAS domain that binds oxygen to inactivate the c-di-GMP-cleaving EAL domain (Chang et al., 2001). Oxygen binding leads to increased c-di-GMP and BC levels (Chang et al., 2001). The *K. xylinus* DGC, AxDGC2, contains a flavin cofactor-binding PAS domain (Qi et al., 2009). Non-covalent binding of FAD to AxDGC2 induced higher catalytic activity compared to FADH2, demonstrating a role of cellular redox status in BC biosynthesis (Qi et al., 2009). Regeneration of FAD primarily occurs at the electron transport chain which uses oxygen as a terminal electron acceptor. Oxygen is required for effective activation of AxDGC2 and inhibition of AxPDEA1, resulting in increased c-di-GMP and BC levels. In nature, *K. xylinus* colonizes the surface of fruits which provides an oxygen-rich environment.

In addition to DGCs and PDEs, the *cdg1* operon also encodes two transcriptional regulators: *cdg1a* and *cdg1d* (Tal et al., 1998). Over-expression of CDG1A results in increased BC production. The *cdg1a* gene encodes a CRP/FNR transcription factor (TF; Tal et al., 1998). Deng et al. (2013) recently identified another CRP/FNR TF gene (GXY_00863) required for BC biosynthesis in *K. hansenii*, a colonizer of fruit and close relative of *K. xylinus*. GXY_00863 is essential for BC production through transcription of *bglAx* and other unidentified genes required for BC biosynthesis. The role of CDG1D in BC biosynthesis has not been demonstrated, but sequence analysis reveals that it belongs to the Rrf2 repressor family of transcriptional regulators, therefore it may repress BC biosynthesis through a yet to be discovered mechanism.

#### The Role of Environmental Acidification in Fruit Colonization

*Komagataeibacter xylinus* secretes large quantities of various organic acids (Zhong et al., 2013, 2014). Although production of organic acids drains carbon away from *K. xylinus* BC production (Zhong et al., 2013, 2014), substrate acidification serves an ecological purpose. In the context of fruit–bacteria interactions, environmental acidification through secretion of organic acids provides *K. xylinus* with a competitive advantage over less acid-tolerant organisms growing on the same substrate. This phenomenon has been observed with various vaginal lactobacilli and oral streptococci strains that acidify their environment to outcompete competitors (Quivey et al., 2000; Graver and Wade, 2011). Kawano et al. (2002) demonstrated that *K. xylinus* ATCC 53582, but not *K. hansenii* ATCC 23769 synthesizes BC after glucose depletion by utilizing gluconic acid as a carbon source. While gluconic acid production is correlated with decreased pH, gluconic acid metabolism increases culture pH (Kawano et al., 2002). In the context of fruit–bacteria interactions, strains of *K. xylinus* acidify their microenvironment through gluconic acid secretion until they become the predominant organism on the fruit substrate. Once established, *K. xylinus* resorbs gluconic acid for BC production to enhance colonization. Secretion of other acids, particularly acetic acid, may contribute to this process. Environmental acidification also solubilizes essential micronutrients such as phosphate and iron as previously reported for other plant-associated bacteria, including *Gluconacetobacter diazotrophicus*, *B. subtilis*, *Pseudomonas*, and *Rhizobium* species (Rodríguez and Fraga, 1999; Zhang et al., 2009a; Crespo, 2011).

#### Influence of Plant Cell Wall Components (PCWCs) on BC Structure

*In vitro* and *in planta* biofilm production by the Gram-positive rhizosphere bacterium, *B. subtilis*, is induced by the plant cell wall polysaccharides arabinogalactan, pectin and xylan (Beauregard et al., 2013), while monosaccharides have no effect. Though the *B. subtilis* biofilm has not been shown to contain BC, it does contain EPS, including the fructans levan I and II (Abdel-Fattah et al., 2005; Dogsa et al., 2013) and another polysaccharide containing glucose, galactose, fucose, glucuronic acid, and *O*-acetyl groups in a molar ratio of about 2:2:1:1:1.5 (Morita et al., 1979). Therefore, plant cell wall components (PCWCs) can have a profound effect on polysaccharide-containing biofilms. Once deposited on fruit by an insect vector (see Insect–bacteria Interactions), *K. xylinus* would encounter several PCWCs that would interact and structurally modify its BC, impacting its ability to anchor itself to the fruit surface as highlighted below. The effect of PCWCs on the genetic regulation of BC has not yet been investigated.

##### BC-hemicellulose

Hemicelluloses are a group of heterogeneous polysaccharides that function as supporting material in plant cell walls where they bind directly and indirectly to cellulose and pectins (Rizk et al., 2000; Zykwinska et al., 2005). The mechanisms involved in the binding of hemicellulose to cellulose during plant cell wall assembly (see review: Cosgrove, 2005) may be used to infer interactions between hemicellulose and BC when *K. xylinus* is growing on fruit. Typically, H-bonds between hemicellulose side chains and cellulose allow for their association (Fry, 1988; Chen, 2014).

*BC-xylan*. The hemicellulose xylan (**Figure 1C**) has a backbone of β-1,4-linked D-xylose with variable numbers of glucuroyl, acetyl and arabinosyl groups typically at the O-2 or O-3 position (Ebringerová and Heinze, 2000). Tokoh et al. (1998) showed that the addition of xylan to the bacterial growth medium decreased the ratio of cellulose Iα to Iβ and discontinuously affected the crystalline structure of BC microfibrils. Xylan incompletely coated the surface of BC bundles or ribbons, revealing striations, and rough surfaces (Tokoh et al., 1998). Xylan affects microfibrils to the same extent as carboxymethylcellulose (CMC), and likely at a similar stage of crystallization (Tokoh et al., 1998). Park et al. (2014) showed that xylan reduced BC crystallinity to a greater extent compared to xyloglucan. When present during BC synthesis, glucuronoxylan, *O*-acetyl-glucuronoxylan, arabinoglucuronoxylan, or arabinoxylan disrupted BC microfibril assembly, altering the crystal structure (Uhlin et al., 1995; Iwata et al., 1998; Tokoh et al., 1998; Winter et al., 2006).

*BC-xyloglucan*. Xyloglucan (**Figure 1D**) is the best studied example of hemicellulose interaction with BC. It is composed of a β-1,4-linked D-glucopyranose backbone, with α-D-xylosyl residues and various other side chain substituents (Schultink et al., 2014). Hanus and Mazeau (2006) propose that the interaction between xyloglucan and BC is based on structural similarity between the xyloglucan backbone and the BC chain. This robust network with BC is dependent on the stage of BC formation, particularly crystallization, as xyloglucan in the growth medium has little effect on pre-existing BC (Gu and Catchmark, 2012). This phenomenon has also been observed in regards to plant cell wall assembly (Baba et al., 1994; Hayashi et al., 1994). The presence of xyloglucan in growth medium greatly reduced Iα allomorph content with a relatively small effect on crystallinity (Park et al., 2014) due to the increased Iβ content in xyloglucan-BC (Hackney et al., 1994; Whitney et al., 1995; Yamamoto et al., 1996; Park et al., 2014). Uhlin et al. (1995) proposed that xyloglucan and xylan co-crystallize with BC by creating lattice defects, thus altering the structure.

*BC-glucomannan*. The hemicellulose with the greatest affinity for BC is glucomannan (**Figure 1E**), a water-soluble polysaccharide that contains β-1,4-linked D-mannose and D-glucose in a ratio of 8:5 (Yui et al., 1992; Iwata et al., 1998). Mannan-based hemicelluloses lower the crystallinity of BC by decreasing the crystallite size in a similar manner as CMC (Uhlin et al., 1995). Whitney et al. (1998) observed extensive heterogeneous structures when glucomannan was in the growth medium with *K. xylinus* BC. The BC acts as a template, resulting in a proportion of the mannose residues in glucomannan adopting a cellulosic confirmation (Whitney et al., 1998). Through ^13^C-NMR analysis, glucomannan was shown to decrease the abundance of Iα allomorph by increasing the disorder in BC, rather than simply shifting the Iα/Iβ ratio of BC produced by *K. xylinus* as is the case with many of the hemicelluloses (Hackney et al., 1994). Thus, hemicellulose from the fruit cell wall could incorporate into BC synthesized by *K. xylinus*, decreasing its crystallinity. In turn, this would increase water absorption by BC, providing protection against desiccation. Additionally, the BC biofilm could coalesce with hemicellulose in the fruit cell wall, thereby serving as an anchor to the fruit.

##### BC-pectin

The primary cell wall of dicotyledons contain approximately 35% pectin and fruits can contain substantially higher amounts (Fry, 1988). *K. xylinus* would therefore be exposed to pectic substances when colonizing fruit. Pectins are hydrophilic PCWCs that are composed of as many as 17 different monosaccharides with over 20 different linkages, making it one of the most complex macromolecules in nature (Ridley et al., 2001; O’Neill et al., 2004; Voragen et al., 2009). Pectic substances include negatively charged homogalacturonans, rhamnogalacturonan I including neutral sugar side chains, rhamnogalaturonan II, and xylogalacturonan (Chanliaud and Gidley, 1999). In the plant cell wall, pectin domains are thought to be connected though covalent linkages, borate esters, and Ca^2+^-mediated ionic cross-linkages (Vincken et al., 2003). Negatively charged carboxylic acid groups of galacturonic acid residues (**Figure 1F**) on adjacent pectin chains are stabilized by Ca^2+^ and form a gel-like substance. Ca^2+^-mediated ionic cross-linkages are prevented by methyl-esterification of galacturonic acid carboxyl groups since they are replaced with a neutral methyl-ester moiety. Consequently, increased methyl-esterification and decreased Ca^2+^ levels weaken the pectin matrix within the plant cell wall (Muller et al., 2012).

Composites of pectin and *K. xylinus* BC form pectin-BC networks which are dependent on the degree of pectin methyl-esterification (DPME) and the Ca^2+^ concentration in the medium (Chanliaud and Gidley, 1999). Pectin-BC association increases linearly until a Ca^2+^ concentration of 8.4 mM, wherein association levels plateau. At this Ca^2+^ concentration, the optimal DPME is 30%. Pectin and BC phase-separate when the DPME is below 30%, resulting in no association. However, as the DPME increases above 30%, pectin-BC association decreases. Under these association conditions, the pectin matrix is established, but pectic chains are not completely fixed by ionic cross-linkages, allowing for pectin and BC chains to associate through electrostatic interactions, H-bonding, and ester bond formation (Fry, 1988; Brett and Waldron, 1996; Chen, 2014). *K. xylinus* colonizes fruit during the ripening stage which is accompanied by activation of pectinases that structurally modify the pectic network in the fruit cell wall. These modifications include solubilization, depolymerization, loss of neutral side chains, and overall weakening (Prasanna et al., 2007; Paniaqua et al., 2014). *K. xylinus* BC would therefore associate with the weakened pectin network in the fruit cell wall during colonization. BC formed by *K. xylinus* in nature would be expected to incorporate pectins from the fruit cell wall, increasing recalcitrance and aiding its ability to anchor to the substrate.

##### BC-lignin

Aromatic polyphenolic lignin is also present in the plant cell wall. Lignin is unique because its synthesis is due to free-radical condensation of *p*-coumaryl alcohol (**Figure 1G**), coniferyl alcohol (**Figure 1H**), and sinapyl alcohol (**Figure 1I**) rather than strict enzymatic control (Sarkanen and Ludwig, 1971) leading to an undefined ultrastructure. Lignin is primarily responsible for the recalcitrance of lignocellulosic biomass (Zeikus et al., 1982; Donaldson, 2001). Lignin and various polysaccharides interact through electrostatic dipole–dipole interactions responsible for polysaccharide–lignin and pectin–lignin association in the plant cell wall (Sugino et al., 1994; Houtman and Atalla, 1995). Lignin is likely to incorporate into the BC biofilm of *K. xylinus* during fruit colonization. The resulting bacterial lignocellulose (BLC) complex would be more recalcitrant than BC alone, similar to how lignin increases the strength of plant cell walls by incorporating into PC. A biofilm consisting of BLC would provide a distinct advantage to *K. xylinus* over BC non-producers colonizing fruit, particularly against cellulase-producing microbes, while simultaneously improving attachment and anchoring of the BC-containing biofilm to the fruit.

**FIGURE 6:**
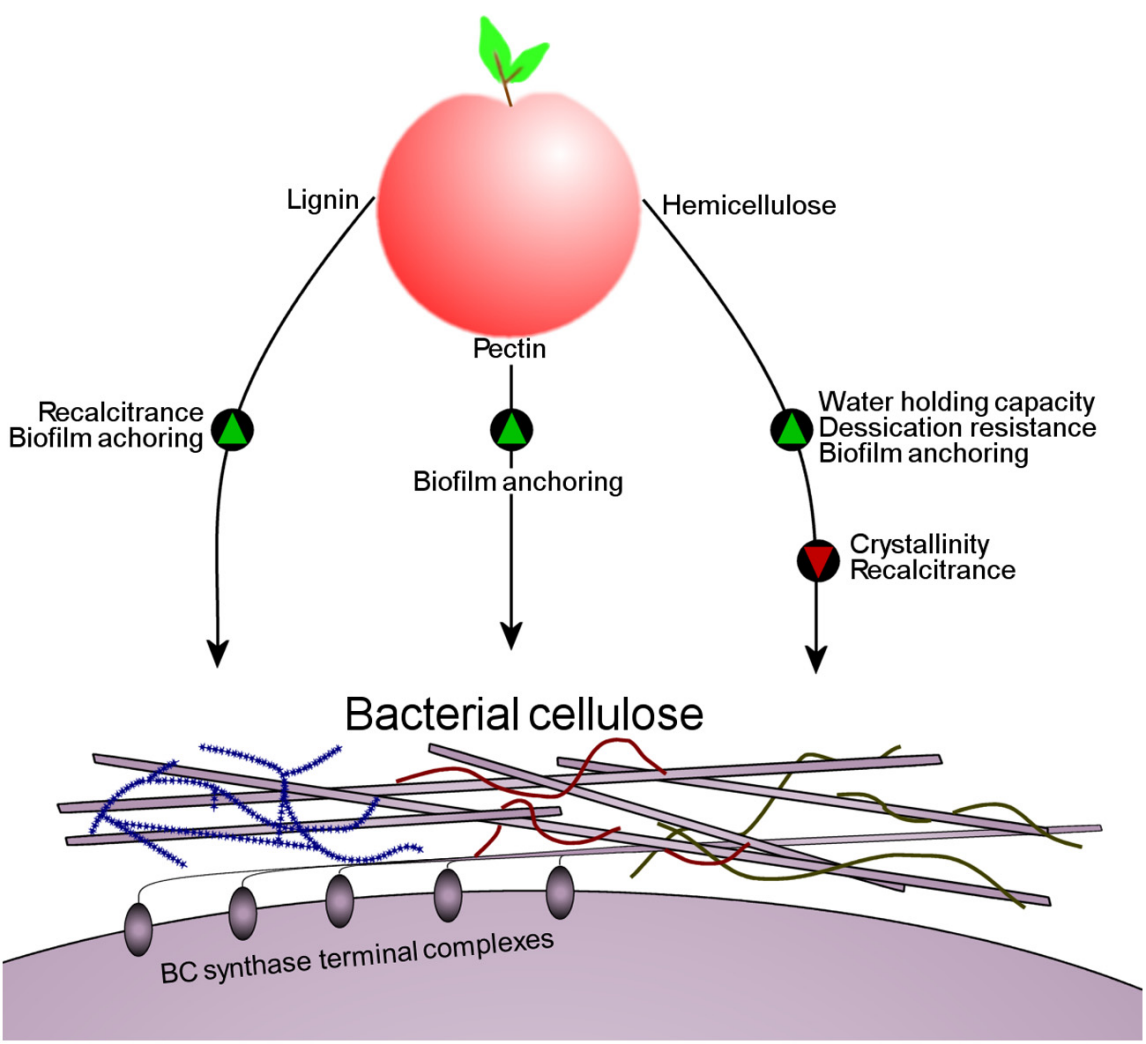
**A schematic illustrating the effect that the PCWCs lignin, pectin and hemicellulose have on the structure of BC when *K. xylinus* colonizes fruit.** Green triangles indicate that a characteristic would be promoted, while red triangles indicate that a characteristic would be reduced. All PCWCs associate with the BC enhancing anchoring to and colonization of the fruit by bacteria.

Observations that BC associates with PCWCs makes it clear that hemicellulose, pectin and lignin in the plant cell walls affect the structure of *K. xylinus* BC influencing its colonization of fruit. Hemicelluloses incorporated into BC affect ribbon assembly and reduce crystallization (see above), identical to the effects of bacterially produced HE-EPS on BC (Fang and Catchmark, 2014; Deng et al., 2015). Though decreasing BC crystallinity through hemicellulose incorporation may make the *K. xylinus* biofilm more susceptible to enzymatic degradation, incorporation of lignin into the BC matrix to form BLC would simultaneously increase its recalcitrance and permit water retention, an advantage against desiccation. Incorporation of hemicelluloses into the BC matrix would mask the BC microfibrils protecting them from environmental cellulases. Association between PCWCs and BC would allow *K. xylinus* to firmly anchor itself to the fruit substrate through direct interactions of the fruit cell wall and BC. To our knowledge, this adherence mechanism has not been previously proposed, but may be involved in the colonization process of other plant-associated bacteria. A proposed model of this interaction based on available data is shown in **Figure 6**.

#### Effects of Plant-derived Compounds (PDCs) on BC Biosynthesis

In addition to PCWCs, *K. xylinus* would be exposed to other plant-derived compounds (PDCs) on fruit that could influence BC biosynthesis, its regulation and its ultrastructure which in turn would impact fruit colonization.

##### Ascorbic acid (AsA, vitamin C)

An example of a PDC shown to affect BC biosynthesis is ascorbic acid (AsA, vitamin C; **Figure 1J**), which is present at high concentrations in fruits and vegetables (Wimalasiri and Wills, 1983). Keshk (2014) demonstrated that addition of 0.5% (w/w) AsA to the culture medium of four *K. xylinus* strains doubled the BC yield compared to unammended cultures. A decreased gluconic acid concentration concomittant with increased final pH of the culture medium was also observed providing evidence that AsA enhances the ability of *K. xylinus* to use gluconic acid for BC production (Kawano et al., 2002). AsA decreases the crystallinity index of BC by influencing H-bonding between adjacent glucan chains allowing for increased water absorption; therefore more water-soluble AsA can penetrate the BC biofilm. This perpetuates the positive effect that AsA has on BC yield, enhancing colonization of the fruit substrate. The change in AsA concentration during ripening is fruit-specific, but approaches maximal levels at the end of the ripening stage (Mellidou et al., 2012; Muhammad et al., 2014). Therefore, AsA acts as a signaling molecule in nature, informing *K. xylinus* that ripening has commenced, and that nutrients are available for BC production. It should be noted that cell growth was not measured in these studies so it is not known whether the positive effect exerted by AsA on BC production was a direct or an indirect effect of increased growth, as observed for the phytohormones ABA, zeatin, and GA_3_ (Qureshi et al., 2013).

##### Phytohormones

*Komagataeibacter xylinus* can be isolated from fleshy fruit where it would be exposed to various phytohormones that work in concert to control plant growth, development, and interaction with external stimuli. Indole-3-acetic acid (IAA; **Figure 1K**) is an auxin involved in nearly every aspect of plant growth and development (Zhao, 2010). Abscisic acid (ABA; **Figure 1L**) controls root growth, seed dormancy, the opening and closing of stomata, and the adaptive stress response to biotic and abiotic factors (Cutler et al., 2010). Zeatin (**Figure 1M**) is a cytokinin that regulates cytokinesis and differentiation of plant cells, and works in conjunction with IAA to control plant growth (Hitoshi, 2006). Gibberellic acid (GA_3_; **Figure 1N**) encourages plant cells to increase in size through the uptake of photosynthate (Davière and Achard, 2013). Ethylene (**Figure 1O**) is a gaseous phytohormone that interacts with other phytohormones to regulate vegetative development, flowering and ripening (Vandenbussche and Van Der Straeten, 2012).

Fruit growth, characterized by cell division and expansion, is initially signaled by IAA, zeatin and GA_3_ sent from the seed to surrounding tissues, followed later by an increase in ABA. Fruit ripening and senescence is the final developmental stage, wherein levels of IAA, Z, and GA_3_ remain low, and levels of ABA and ethylene increase.

Fruit ripening is associated with numerous physiological and biochemical changes; the sweetness of ripe fruit is caused by starch hydrolysis. ABA promotes this process by up-regulating ethylene production, which increases amylase activity resulting in sugar accumulation (Agravante et al., 1990; Montalvo et al., 2009; Zhang et al., 2009b). The fruit cell wall is weakened during ripening (see review: Brummell, 2006). This process, triggered by ABA and ethylene (Lohani et al., 2004; Cantín et al., 2007), is associated with increased activity of cell wall-degrading pectolytic and cellulolytic enzymes (Ahmed and Labavitch, 1980; Brummell and Harpster, 2001; Lohani et al., 2004). Intense turgor pressure, combined with the weakening of the plant cell wall, releases exudate onto the surface of the fruit, leaving it more susceptible to microbial invasion. Other phytohormones are involved in fruit ripening but are not pertinent to this review.

Accumulation of sugars and weakening of the fruit cell wall during ripening provides a suitable nutrient environment for BC production and colonization. *K. xylinus* participates in numerous plant–microbe interactions, including the bi-directional transfer of phytohormones. While the effect of bacterially produced phytohormones on plant physiology has been well-studied (Costacurta and Vanderleyden, 1995; Spaepen and Vanderleyden, 2011), there is limited information regarding the effect of plant-produced phytohormones on bacterial physiology.

*Indole-3-acetic acid*. The role of IAA in plant–bacteria interactions of rhizosphere bacteria, such as *Gluconacetobacter*, *Pseudomonas*, *Agrobacterium, Rhizobium*, *Bradyrhizobium, Enterobacter*, *Azospirillum*, and *Streptomyces* (Duca et al., 2014) is well-studied. Depending on the amount of IAA secreted and the sensitivity of the plant to IAA, these bacteria can have a plant growth promoting (Glick, 2012), or phytopathogenic effect (Spaepen and Vanderleyden, 2011). For example, crown gall tumor formation by *A. tumefaciens* and *A. rhizogenes* depends on production of auxins and cytokinins that induce rapid cell division in plant roots. Genes for the biosynthesis of IAA, zeatin-riboside and *trans*-zeatin are found on the T-DNA region of the tumor-inducing plasmid (Akiyoshi et al., 1984; Thomashow et al., 1984). Unlike the rhizosphere bacterium *G. diazotrophicus* (Lee and Kennedy, 2000), the closely related carposphere bacterium *K. xylinus*, does not produce endogenous IAA (Qureshi et al., 2013), as it would be counter-productive for its survival; IAA inhibits fruit ripening (Davies et al., 1997; Aharoni et al., 2002).

Exogenous IAA stimulates the growth of *K. xylinus* but diminishes BC yield, suggesting that IAA has a direct effect on BC production (Qureshi et al., 2013). The observed lower BC yield is consistent with the fruit–bacteria interactions of *K. xylinus*, since IAA inhibits ripening and signals monosaccharide building blocks are unavailable for BC synthesis. Delaying BC production preserves carbon source for growth. In nature, *K. xylinus* growth stimulation by IAA ensures cell density is at a peak once fruit ripening begins and IAA levels decrease (Jia et al., 2011). Whether IAA-induced growth is a result of IAA metabolism is unknown. IAA metabolism and degradation (Leveau and Lindow, 2005; Leveau and Gerards, 2008) has been observed in other bacteria and recently reviewed (Duca et al., 2014). It has been suggested that the degradation or inactivation of IAA by *K. xylinus* could decrease its effective concentration, thereby allowing fruit ripening to commence (Qureshi et al., 2013). Two rhizobacteria, *Rhodococcus* sp. P1Y and *Novosphingobium* sp. P6W, metabolize ABA and decrease its concentration *in planta*, altering host–plant growth (Belimov et al., 2014). Lowered BC production in the presence of IAA permits *K. xylinus* to chemotactically find nutrient conditions better suited for colonization (i.e., surface-wounds on fruit).

Indole-3-acetic acid and other indole derivatives act as signaling molecules in bacteria, having effects on biofilm formation (Lee et al., 2007) and quorum sensing (Pillai and Jesudhasan, 2006). Similar to IAA effects on *K. xylinus* BC production, indole (**Figure 1P**) decreases biofilm formation in *E. coli* (Martino et al., 2003; Lee et al., 2007). The effect of indole on biofilm formation was first reported to be dependent on SdiA, an *N*-acyl-homoserine lactone receptor (Lee et al., 2007) but recent data suggests this is not the case (Sabag-Daigle et al., 2012). SdiA belongs to the LuxR family of transcriptional regulators. The genome sequence of *K. xylinus* E25 contains three genes (H845_2765, H845_67, and H845_1915) that encode LuxR family transcription factors which may be involved in *K. xylinus* quorum sensing; an area of research that has yet to be investigated.

*Abscisic acid*. Abscisic acid triggers fruit ripening during the development of climacteric and non-climacteric fruit, though its effect is more pronounced in non-climacteric varieties (Li et al., 2011). Decreased IAA levels, and increased concentrations of ABA during ripening triggers the biosynthesis of ethylene (Zhang et al., 2009b), initiating a cascade of physiological changes that render the fruit more suitable for colonization by *K. xylinus* (see above).

Abscisic acid biosynthesis has been reported in the phytopathogens *Azospirillum brasilense* (Perrig et al., 2007; Cohen et al., 2008) and *Azospirillum lipoferum* (Bottini et al., 2009), various plant-growth promoting bacteria such as *Bradyrhizobium japonicum, Rhizobium* sp., *Proteus vulgaris, Klebsiella pneumoniae, Bacillus megaterium*, and *Bacillus cereus* (Dangar and Basu, 1991; Tuomi and Rosenqvist, 1995; Karadeniz et al., 2006; Boiero et al., 2007) and numerous other endophytic bacteria (Sgroy et al., 2009). *K. xylinus* also produces endogenous ABA (Qureshi et al., 2013), which likely plays a role in its ability to colonize fruit. *A. brasilense* Sp245 produces ABA and increases endogenous ABA levels in *Arabidopsis* (Cohen et al., 2008). ABA biosynthesis by *A. lipoferum* USA 59b stimulates the growth of plants in dry soil (Bottini et al., 2009). Analogously, ABA production by *K. xylinus* could accelerate fruit ripening by stimulating the fruit’s endogenous ABA levels. This encourages plant ethylene production, leading to degradation of PC and starch and the liberation of free glucose providing ideal conditions for fruit colonization. ABA also plays a key role in regulating plant–pathogen interactions. In plants, there is a positive correlation between ABA levels and susceptibility to pathogens (Mohr and Cahill, 2003; Thaler and Bostock, 2004; Fan et al., 2009). ABA production by *K. xylinus* may also serve to down-regulate host–plant defenses.

Qureshi et al. (2013) showed that exogenous ABA enhanced the growth and BC production of *K. xylinus*. Though monosaccharides in an unripe fruit are scarce, a ripe fruit provides the carbon required for growth, BC production and colonization. ABA therefore acts as a signal to *K. xylinus* indicating that the environment is suitable for colonization. In response, *K. xylinus* growth increases as does BC synthesis, enhancing its ability to outcompete other organisms inhabiting the same fruit (Williams and Cannon, 1989).

*Zeatin and gibberellic acid (GA_3_)*. Fruit growth occurs prior to ripening, and is regulated by zeatin and GA_3_, which work with IAA to induce cytokinesis and cell enlargement, respectively. Bacterial biosynthesis of zeatin and GA_3_ is documented in numerous plant growth promoting bacteria (Phillips and Torrey, 1972; Atzorn et al., 1988; Bottini et al., 1989; Bastián et al., 1998; Arkhipova et al., 2005; Karadeniz et al., 2006; Boiero et al., 2007) and bacterial phytopathogens (Akiyoshi et al., 1987; Karadeniz et al., 2006). These bacteria also produce IAA and influence cell division, and growth of host plants. Qureshi et al. (2013) showed that *K. xylinus* produces endogenous zeatin and GA_3_. *B. subtilis*, when grown in association with lettuce, produces zeatin causing an increase in plant shoot and root weight (Arkhipova et al., 2005). GA_3_ production by various root-colonizing bacteria is also accompanied by increased plant growth (see review: Bottini et al., 2004). Inoculation with GA_3_-producing *G. diazotrophicus*, or exogenous GA_3_, increases monosaccharide levels in *Sorghum bicolor* (Bastian et al., 1999). Exogenous GA_3_ also increases the size and sugar content of grapes (Casanova et al., 2009). These studies demonstrate that bacterial zeatin and GA_3_ production can influence plant development.

Exogenous zeatin and GA_3_ increase *K. xylinus* growth and BC production (Qureshi et al., 2013). However, similar to the effects of ABA, the positive effect of zeatin and GA_3_ on BC production is indirect.

**FIGURE 7:**
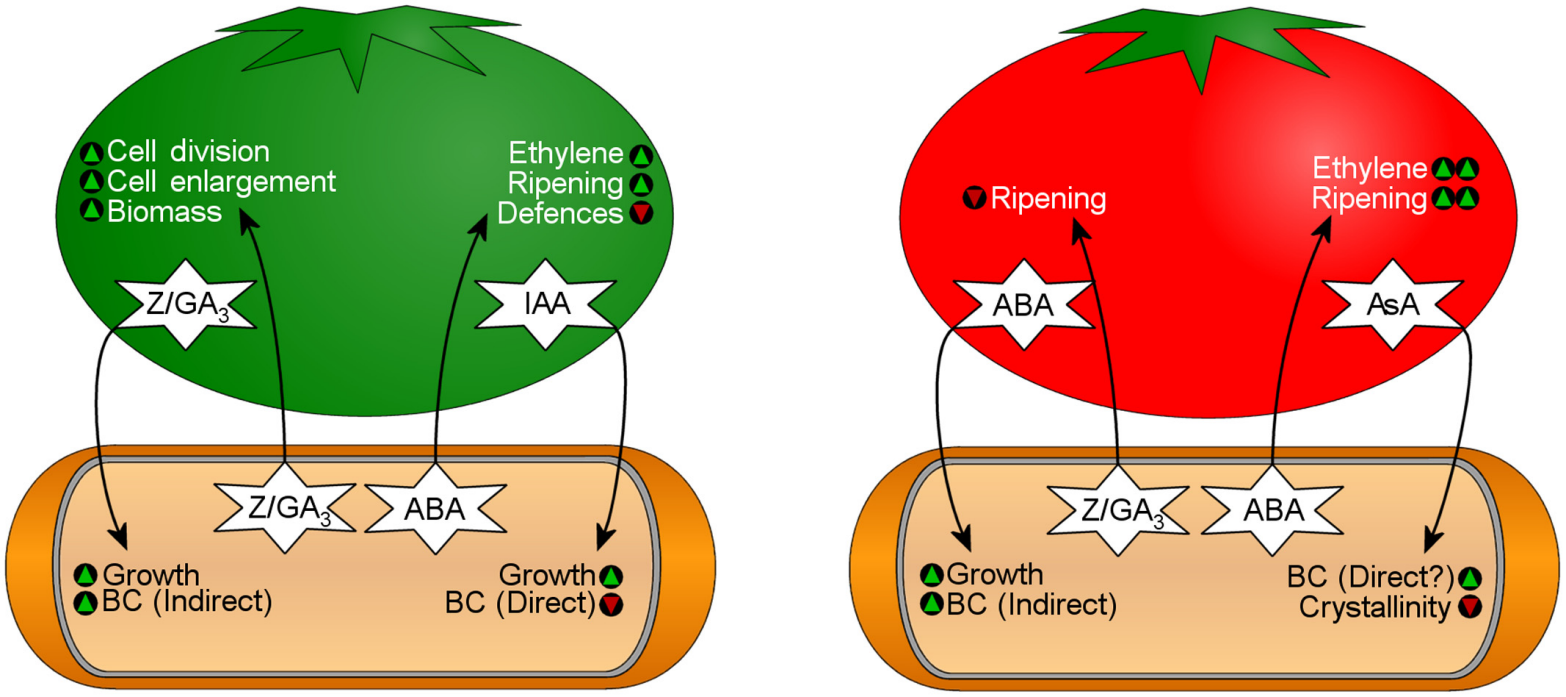
**Bi-directional transfer of phytohormones influences bacterial physiology and fruit development during K. xylinus fruit colonization.** The fruit on the left represents an unripe fruit that contains high levels of indole-3-acetic acid (IAA), zeatin (Z), and gibberellic acid (GA_3_). The fruit on the right represents a ripe fruit that contains high levels of abscisic acid (ABA) and ascorbic acid (AsA). See text for details. Green triangles indicate that a characteristic would be promoted, while red triangles indicate that a characteristic would be reduced.

Based on Qureshi et al. (2013), IAA, zeatin and GA_3_, which are present at high concentrations in unripe fruit, stimulate *K. xylinus* growth ensuring high cell density once ripening begins. IAA directly down-regulates BC biosynthesis, since it is an indicator that fruit is not ripe. Endogenous zeatin and GA_3_ production increases fruit size, providing more biomass for colonization. Higher cell densities enhance endogenous ABA production by *K. xylinus* inducing fruit ripening through the activation of plant-produced ethylene. IAA, zeatin, and GA_3_ levels drop significantly concommitant with an increase in ABA during the ripening stage, a signal to *K. xylinus* that ripening has begun and that carbon source is available for BC production. A proposed model for this process is shown in **Figure 7**.

### Other Plant–bacteria Interactions in the Phyllosphere

In addition to the carposphere, BC producers inhabit other phyllosphere microenvironments. For example, the γ*-proteobacterium P. syringae* pv. *syringae* UMAF0158, the causal agent of bacterial apical necrosis (Cazorla et al., 1998), produces BC (Arrebola et al., 2015) to adhere to the surface of mango and tomato leaves. *P. fluorescens*, also produces BC for its environmental interactions (see review: Spiers et al., 2013).

The γ*-proteobacterium*, *Dickeya dadantii* (formerly *Erwinia chrysanthemi*) is another BC producer inhabiting the phyllosphere (Jahn et al., 2011) that forms pellicles and biofilms containing BC (Yang et al., 2008) which facilitate its broad-host-range phytopathogenicity. The ecophysiology of *D. dadantii* has been discussed in more detail elsewhere (see review: Reverchon and Nasser, 2013).

### Persistence of Pathogenic *Enterobacteriaceae* on Fresh Produce

Human pathogens, such as *E. coli* and *Salmonella enterica*, can form biofilms on fruits and vegetables surviving off exudate released from lysed plant cells. *Enterobacteriaceae* are found on sprouts, green leafy vegetables and fruits, such as melons and tomatoes, where they adhere to, but typically do not cause disease symptoms (Doyle and Erickson, 2008; Niemira and Zhang, 2009). However, *S. enterica* serovar Typhimurium acts as phytopathogen in maize and mung bean plants when inoculated onto non-germinated seeds (Singh et al., 2004, 2005). *S. enterica* serovar Typhimurium and the enterohemorrhagic *E. coli* O157:H7 persists for over 100 days on basil and 177 days on parsley, respectively (Islam et al., 2004; Kisluk et al., 2013). The longevity of pathogens on fresh produce is largely due to EPSs and curli, which contribute to resistance against chlorine washes, a commonly used sanitizer in the food industry (Beuchat, 1997, 1999; Taormina and Beuchat, 1999; Ryu and Beuchat, 2005).

Bacterial cellulose and curli are two main factors in adherence of *Enterobacteriaceae* on produce. *S. enterica* serovar Typhimurium mutants that were unable to produce BC or curli were found to have a one log reduction in adherence to parsley leaves (Lapidot and Yaron, 2009). The role of BC in adherence was also demonstrated by mutations in *bcsA* and *bcsC*, in which *S. enterica* serovar Enteritidis and *S. enterica* serovar Typhimurium had reduced attachment to either alfalfa sprouts and tomato fruits, respectively (Barak et al., 2007; Shaw et al., 2011). Other studies have shown that pre-cut produce increases the risk of contamination, as several *S. enterica* serovars were shown to preferentially attach to cut surfaces of many types of produce (Patel and Sharma, 2010). In some studies, *E. coli* displays less dependence on BC for adherence. The ability for enterohemorrhagic *E. coli* O157:H7 to attach to spinach leaves was shown to be associated with curli rather than BC (Macarisin et al., 2012). The highly virulent and BC non-producing *E. coli* O104:H4 was found contaminating sprouts (Buchholz et al., 2011; Richter et al., 2014). This strain contains a novel hyperactive DGC (GdcX), and over-produced both CsgD and curli (Richter et al., 2014) which, among other factors, may assist with adherence in the absence of BC. However, these studies are not representative for all *E. coli* strains since *E. coli* O103:H2 relies on BC for attachment to lettuce leaf surfaces, but not to their cut edges (Lee et al., 2015).

A review by Yaron and Römling (2014) identified several *Enterobacteriaceae* plant adherence genes that encode virulence factors in animals. In *S. enterica* serovar Enteritidis, these genes include the biofilm regulator *csgD* and the curli nucleator, *csgB* (Barak et al., 2005) suggesting a mechanism in which pathogens could persist in the environment and use fresh produce as vehicles to infect human hosts (see review: Berger et al., 2010).

## Animal–Bacteria Interactions of BC Producers

Bacteria can either have a pathogenic or symbiotic relationship with their animal hosts mediated by BC and biofilm formation. Especially interesting is the ecological diversity of BC biofilm regulation and the role of BC in the colonization of animal hosts as highlighted below.

### Pathogenesis of BC-producing *Enterobacteriaceae* in Humans

Many *Enterobacteriaceae* produce BC-containing biofilms. Two of these γ*-proteobacteria* are the well-studied foodborne pathogens, *E. coli* and *S. enterica.* The biofilms produced by these bacteria are highly heterogeneous with a mixture of polysaccharides, such as BC and colanic acid, in addition to proteinaceous components, such as curli fimbriae (Grant et al., 1969; Zogaj et al., 2001; Solano et al., 2002). BC is an essential component of these biofilms, as the loss of BC production in some *Enterobacteriaceae* prevents adhesion, biofilm maturation, and multicellular behavior (Da Re and Ghigo, 2006; Lapidot and Yaron, 2009). Colanic acid, on the other hand, does not improve adhesion but does appear to function in biofilm maturation (Danese et al., 2000; Prigent-Combaret et al., 2000; Hanna et al., 2003; Ledeboer and Jones, 2005). Similar to BC, synthesis of curli has been associated with improved adherence to animal hosts (Prigent-Combaret et al., 2000; Barak et al., 2005). *E. coli* produces an additional polysaccharide composed of a peptidoglycan subunit *N*-acetylglucosamine (GlcNAc) to form poly-β-1,5-GlcNAc (PGA) that aids in attachment and biofilm integrity (Wang et al., 2004; Itoh et al., 2005) underscoring the importance of the biofilm lifestyle for this pathogen.

#### Transcriptional Regulation of BC and Curli Biosynthesis

*Escherichia coli* and *S. enterica* serovar Typhimurium share major biofilm regulatory mechanisms responsible for curli and BC synthesis through the key biofilm regulator CsgD (sometimes named AgfD in *S. enterica*), which belongs to the FixJ/LuxR-family of transcription regulators (Römling et al., 1998; Pedersen et al., 2000; Zogaj et al., 2001). In *S. enterica* serovar Typhimurium, the expression of CsgD is controlled by environmental conditions through the stress response sigma factor RpoS via the regulator MlrA (Brown et al., 2001; Gerstel and Römling, 2001, 2003). Both *rpoS* and *csgD*, act as central activators for multiple environmental signals via small RNAs (see review: Mika and Hengge, 2014). CsgD stimulates curli production through transcriptional regulation of *csgBAC* and *csgDEFG* (sometimes named *agfBA* and *agfDEFG* in *S. enterica*) operons which encode genes for curli fimbriae (Grantcharova et al., 2010). In *Salmonella* sp., these have been named aggregative fimbriae (*agf*) genes, uniquely from the *E. coli* curli subunit genes (*csg*). The use of *csg* and *agf* designations have become synonymous in *Salmonella.* Therefore, we suggest a consistent nomenclature for *csgD*, *csgBA, csgDEFG*, and respective genes replacing the use of *agfD*, *agfBA*, and *agfDEFG*. BC biosynthesis is regulated by the DGC gene *adrA* (previously *yaiC*), which is present in both *E. coli* and *S. enterica* and promoted by CsgD (Brombacher et al., 2003; Simm et al., 2004). AdrA activates BcsA through the synthesis of the c-di-GMP. Although CsgD is a key promotor for biofilm production, it also induces the expression of the PDE gene, *yoaD* (Brombacher et al., 2006). Elevated YoaD expression leads to decreased cell aggregation, while repression leads to increased cellulose production (Brombacher et al., 2006) underscoring the complex role that CsgD plays in the regulation of BC biosynthesis.

**FIGURE 8:**
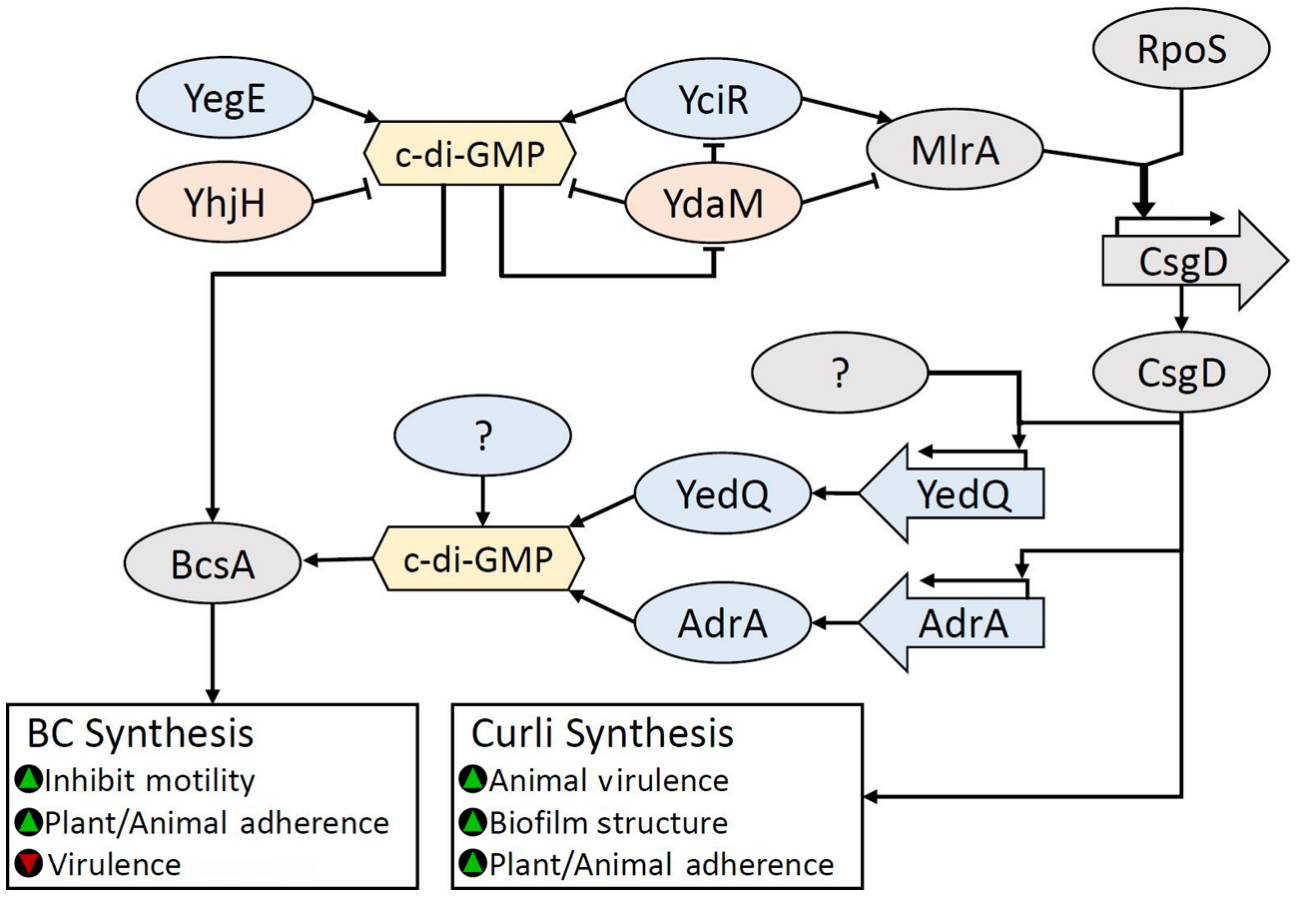
**Regulation of BC biosynthesis in *Enterobacteriaceae.*** BC biosynthesis is regulated by c-di-GMP through the action of many diguanylate cyclases (DGCs). Accumulation of c-di-GMP inhibits the multifunctional phosphodiesterase (PDE) YdaM, which breaks down c-di-GMP, and inhibits both the DGC YciR and transcription factor (TF) MlrA through direct interaction. YciR produces c-di-GMP but also stimulates MlrA activity through direct interaction. High c-di-GMP levels allow MlrA to dramatically increase RpoS induced CsgD expression. RpoS and CsgD are key regulators for biofilm regulation. CsgD-induced expression of AdrA is the primary pathway for BC biosynthesis, however DGCs that are either dependent or independent of CsgD for activation, such as the DGC YedQ have been identified. It is likely that other c-di-GMP pools, such as the YegE/YciR formed c-di-GMP, may also contribute to BcsA activation. Green triangles indicate that a characteristic would be promoted, while red triangles indicate that a characteristic would be reduced.

The pathway involving MlrA, CsgD, and AdrA is known as the CsgD-dependent BC biosynthesis pathway. However, two CsgD-independent pathways have been identified in *E. coli* 1094: the DGC YedQ-dependent and the YedQ-independent pathway (Da Re and Ghigo, 2006). Da Re and Ghigo (2006) showed that mutation of the *E. coli* 1094 *rpoS* gene led to BC negative colonies, but found that the *rpoS* mutation did not significantly affect the expression of *yedQ*, suggesting an RpoS/CsgD-independent regulatory mechanism for *yedQ.* In light of these results, RpoS is likely the highest tier regulator for biofilm production in most *Enterobacteriaceae*. All BC biosynthesis pathways in *Enterobacteriaceae* converge at the c-di-GMP-activated BC synthase, BcsA. This complex regulatory system is summarized in **Figure 8**.

#### Effect of Animal–bacteria Interactions on BC Production

Environmental signals for the expression of *csgD* include nutrient availability, oxygen tension, temperature, pH, ethanol, osmolarity, and acid (see review: Gerstel and Römling, 2003). Under acidic conditions, such as when *Enterobacteriaceae* pass through the acidic gastrointestinal tract during infection, transcription of *csgD* is promoted through the acid-stress response regulator RstA (Ogasawara et al., 2010), stimulating BC production and facilitating attachment to host cells. Additional regulatory genes include the global transcriptional regulator gene o*mpR*, another *csgD* activator, which is responsible for decreased *E. coli* virulence in a *Drosophila* model (Prigent-combaret et al., 2001; Pukklay et al., 2013). In *Drosophila*, virulence is mitigated through EnvZ-OmpR (Yang and Inouye, 1991; Cai and Inouye, 2002; Jubelin et al., 2005; Pukklay et al., 2013). A high-salt medium represses *csgD* expression when CpxR occupies the OmpR promoter site (Jubelin et al., 2005). Inversely, *S. enterica* represses BC production to increase virulence in macrophages through the *mgtC* gene, which downregulates c-di-GMP and *bcsA* (Pontes et al., 2015). Combined, these findings illustrate the complex role of BC biofilms in animal–bacteria pathogenesis and the balance between virulence and long-term viability within a host.

Depending on the strain, BC either serves an essential role in, or is independent of adhesion to animal host tissues. For instance, BC is essential for the probiotic *E. coli* strain, Nissle 1917 to attach to gastrointestinal epithelial cell lines (HT-29) and mouse epithelium (Monteiro et al., 2009). In contrast, the commensal *E. coli* TOB1 has no dependence on BC for adherence to HT-29 cells, and co-expression of BC with curli decreased adherence to HT-29 cells (Wang et al., 2006). In two pathogenic strains of *E. coli*, a single mutation in the key biofilm regulator *csgD* or BC synthase gene, *bcsA* had no significant effect on either cow or human colon tissue cell adherence. However, the *bcsAcsgA* double mutant displayed significantly less adherence than the single mutants or wild type strains (Saldaña et al., 2009). Although adherence to tissue could be compensated by the presence of either gene due to the adherent properties of curli, the three-dimensional biofilm structure was altered as has been observed for curli and BC mutant *S. enterica* serovar Enteritidis strains (Jonas et al., 2007). The transition from a motile to an adherent state is associated with inhibition of flagellar motor proteins, facilitated by activation of the YcgR effector protein via binding of c-di-GMP (Paul et al., 2010). YcgR mediated inhibition of motility occurs by altering the protein–protein interactions of flagellar subunits, FliG and FliM (Fang and Gomelsky, 2010). High levels of c-di-GMP in the absence of YcgR can completely inhibit motility due to the production of cellulose in *S. enterica* serovar Enteritidis (Zorraquino et al., 2013). The use of c-di-GMP insensitive *ycgR* mutants demonstrated that BC produced by neighboring cells did not affect motility. Waters (2013) suggested that inhibition of motility is due to a flagellar “wheel lock,” wherein BC is secreted in close proximity to a cell’s own flagella, mechanically binding it and consequently preventing its rotation. Motility was recovered when the putative endoglucanase gene *bcsZ* was over-produced in the *ycgR* mutants, indicating BC can be hydrolyzed as a quick release mechanism to regain motility (Zorraquino et al., 2013).

Aya Castañeda et al. (2015) demonstrated that regulation of biofilm formation in *S. enterica* was dependent on DAM methylation. Mutants lacking the *dam* gene were unable to produce substantial BC or curli and had reduced *csgD* and *csgA* (major structural subunit of curli fimbriae; Aya Castañeda et al., 2015) but were unaffected in *bcsA* expression suggesting a link between DAM methylation and activation of BC biosynthesis by c-di-GMP. The discovery that DAM methylation affects biofilm gene expression is a novel avenue that warrants investigation in all biofilm producers.

### Insect–bacteria Interactions

Many BC-producing acetic acid bacteria (AAB) are secondary symbionts of insects that rely on sugar-based diets (nectars, fruit sugars, phloem sap), particularly those belonging to the orders Diptera, Hymenoptera, and Hemiptera (see review: Crotti et al., 2010). These α*-proteobacteria* provide their insect hosts with competitive advantages in the environment (Feldhaar and Gross, 2009). *Acetobacteraceae* are naturally found in association with the plants that these insects feed upon facilitating environmental acquistion. The fruit fly *Drosophila melanogaster* and the pink sugarcane mealybug *Saccharicoccus sacchari* have a rich AAB microbiome consisting of *A. aceti*, *G. diazotrophicus*, and *Gluconacetobacter sacchari* (Ashbolt and Inkerman, 1990; Corby-Harris et al., 2007). The BC-producing *Asaia bogorensis* (Kumagai et al., 2011) was originally isolated from the nectar of tropical flowers (Yamada et al., 2000) and subsequently from the gut of insects (Crotti et al., 2009).

Acetic acid bacteria typically inhabit the digestive system, salivary glands and reproductive organs of insects (Dillon and Dillon, 2004), but have also been isolated from insect surfaces (Ren et al., 2007). In the insect digestive tract, AAB flourish due to the aerobic environment, acidic pH and a plethora of diet-derived sugars which are ideal conditions for BC production. Transmission electron microscopy (TEM) has demonstrated that *Asaia* sp. and *Acetobacter tropicalis* embed themselves within the BC matrix to establish tight association to the host epithelium (Favia et al., 2007; Kounatidis et al., 2009). Consistent with observations from the human gut microbiome (see review: Kau et al., 2012), the immune system of *Drosophila* is influenced by its AAB symbionts. For example, the normal microflora of *Drosophila* supresses the proliferation of the pathogenic commensal gut bacterium, *Gluconobacter morbifer* (Ryu et al., 2008).

In addition to being a phytopathogen, BC-producing *D. dadantii*, is also an insect pathogen, as it produces entomotoxins and causes septicemia and death in the pea aphid, *Acyrthosiphon pisum* (Costechareyre et al., 2012). Facilitated by BC production, *D. dadantii* colonizes the gut and forms dense clusters. *A. pisum* acquires bacteria from contaminated plant leaves serving as a vector of *D. dadantii* and phytopathogenic and BC-producing *P. syringae* (Stavrinides et al., 2009).

**FIGURE 9:**
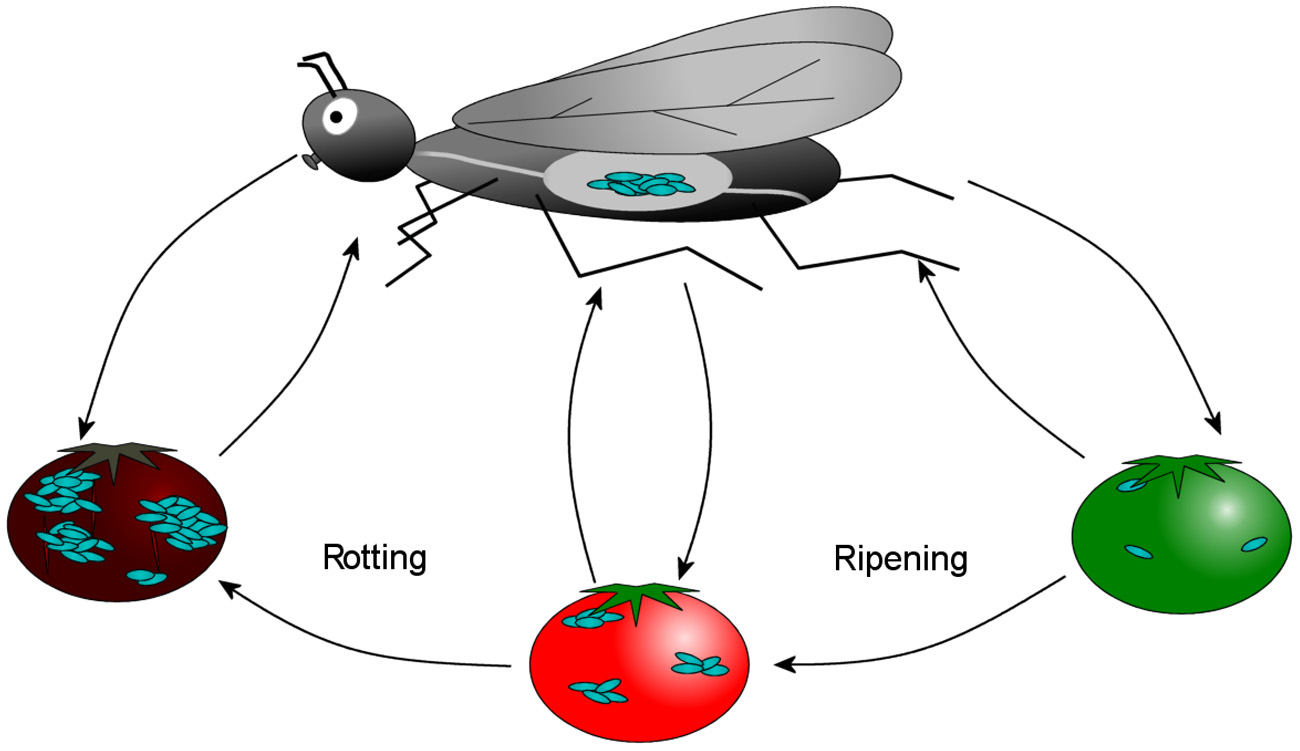
**Sugar-loving insects, such as the fruit fly *Drosophila melanogaster*, acquires and deposits acetic acid bacteria (AAB) onto fruit in nature.** AAB residing in the gut of the insect get deposited on the fruit and encourage ripening, perpetuating this positive feedback loop. This process facilitates the fruit-fruit transmission of AAB that inhabit the carposphere, such as *K. xylinus*.

Since the insect gut serves as a reservoir of AAB, sugar-loving insects act as vectors dispersing various BC-producers to plants in nature. For example, *Drosophila* could deposit and acquire AAB from unripe, ripe or rotten fruit and be responsible for their fruit–fruit transmission (**Figure 9**). This phenomenon has been demonstrated experimentally with non-pathogenic *E. coli* ATCC 11775 and human pathogenic *E. coli* O157:H7. Little is known about how the regulation of BC biosynthesis is affected by insect–bacterium relationships.

### Colonization of the Light Organ of Squid by *Aliivibrio fischeri*

**FIGURE 10:**
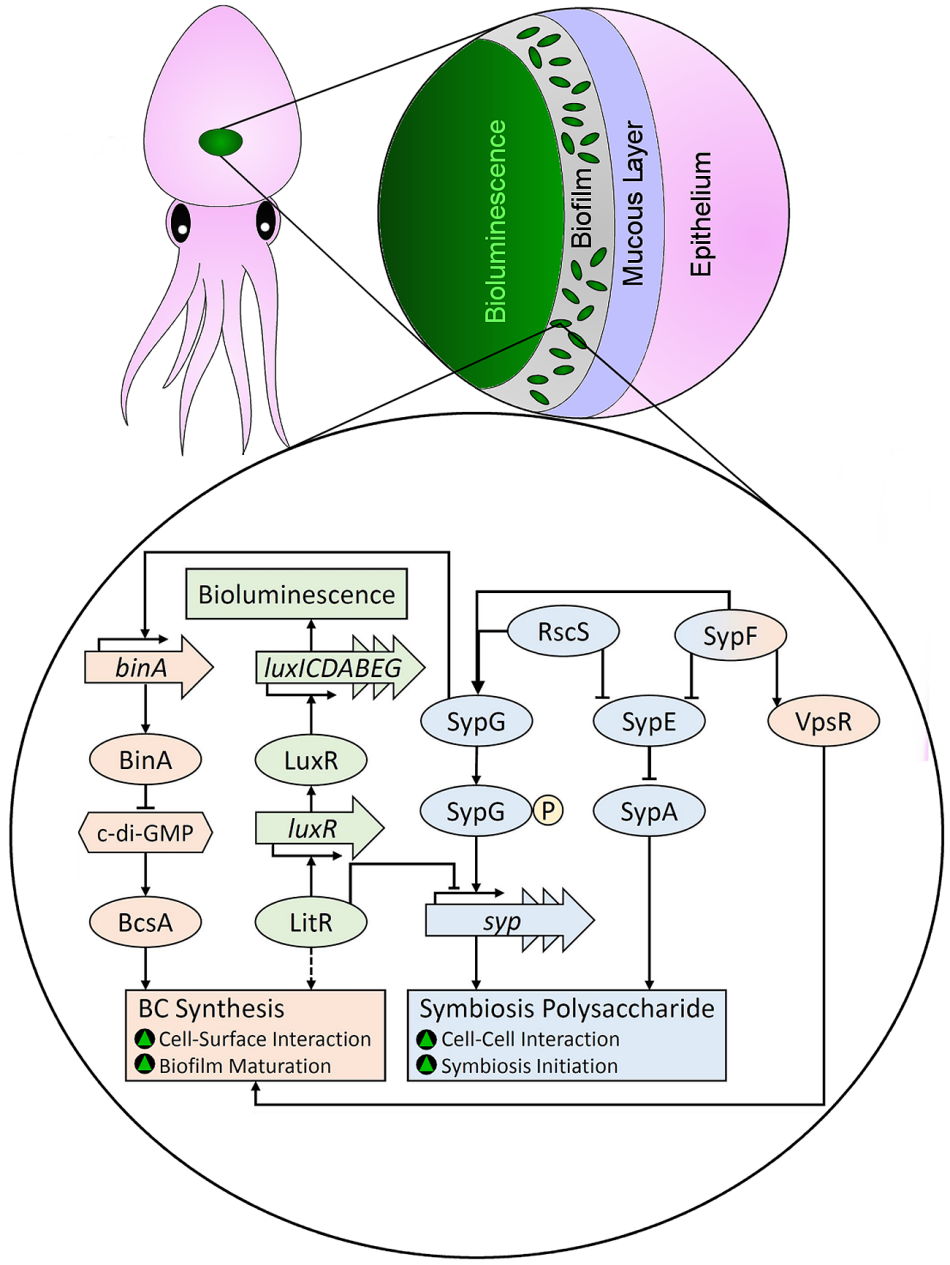
**Regulatory mechanisms responsible for production of BC and the symbiosis polysaccharide (SYP) when *Aliivibrio fischeri* colonizes the light organ of squid.** Pathways in red are involved with BC biosynthesis, pathways in blue are involved with SYP biosynthesis, and pathways in green are responsible for bioluminescence. Green triangles indicate that a characteristic would be promoted. The dashed arrow shows a proposed pathway.

In aquatic environments, γ*-proteobacteria* belonging to the *Vibrionaceae* family form symbiotic or pathogenic relationships with an array of eukaryotic hosts. BC-producing *Aliivibrio fischeri* (formerly *Vibrio fischeri*) participates in a unique symbiotic relationship with the Hawaiian bobtail squid, *Euprymna scolopes*, in which it colonizes and causes bioluminescence in the *E. scolopes* light organ. Light production is controlled by the LuxR–LuxI quorum sensing system which regulates the *luxICDABEG* operon and the luciferase enzyme (Verma and Miyashiro, 2013). Peptidoglycan from the bacterial cell wall induces mucus secretion from the light organ’s ciliated epithelium, which provides the substrate that *A. fischeri* can colonize through biofilm formation (Nyholm et al., 2002). The following section has been summarized in **Figure 10**.

#### The Role and Regulation of the Symbiosis Polysaccharide (SYP)

Colonization of *E. scolopes* by *A. fischeri* relies on biofilm formation. Although pili have been implicated in attachment of pathogenic *Vibrionaceae* to their host (see review: Yildiz and Visick, 2009), it has not been reported for *A. fischeri*. Initiation of squid symbiosis requires a cluster of 18 symbiosis polysaccharide (*syp*) biosynthesis genes that are regulated by RscS (regulator of symbiotic colonization sensor) and σ54-dependent SypG (Yip et al., 2005, 2006). RscS acts upstream of SypG, which is the direct activator of *syp* transcription and production of the SYP. The structure of the SYP has not been fully elucidated, but it does contain glucose and mannose (Yip et al., 2006). Hansen et al. (2014) demonstrated that pathogenic *A. salmonicida* has genes homologous to the *A. fischeri syp* cluster that are transcriptionally repressed by LitR, a master regulator of quorom sensing. LitR transcriptionally activates *luxR*, resulting in bioluminescence (Fidopiastis et al., 2002). Insertional inactivation of *litR* resulted in increased light organ symbiosis, likely through the relief of the repression of *syp* genes. Therefore, LitR coordinates the production of light and SYP, so that bioluminscence occurs in the later stages of colonization. This is consistant with the observation that SYP acts to initiate symbiosis (Shibata et al., 2012). Similar to *A. fischeri*, *A. salmonicida* LFI1238 contains BC biosynthesis genes (Römling and Galperin, 2015).

The activity of another response regulator, SypE, is governed by its phosphorylation state (Morris et al., 2011); unphosphorylated SypE inhibits biofilm formation, while phospho-SypE promotes biofilm formation. SypE controls SypA, a sulfate transporter and anti-sigma antagonist (STAS) domain protein, which is a positive effector of biofilm formation (Morris and Visick, 2013). SypA promotes biofilm formation by a yet to be discovered mechanism when dephosphorylated. SypF, another sensory kinase, controls the activity of SypE, and promotes biofilm formation through a SypE/SypG-dependent mechanism (Darnell et al., 2008). In addition, biofilm induction by SypF requires VpsR, a response regulator that also activates BC biosynthesis (Darnell et al., 2008).

#### The Role and Regulation of *A. fischerii* BC Biosynthesis

Bacterial cellulose biosynthesis is required for the maturation of *A. fischeri* biofilms in the later stages of colonization (Bassis and Visick, 2010). Production of the BC EPS (VPS in *Vibrionaceae*) is controlled by the response regulator VpsR (Darnell et al., 2008), which is homologous to CsgD in *Enterobacteriaceae*. Disruption of *vpsR* results in reduced symbiotic initiation, confirming a role of BC in this process (Hussa et al., 2007). SypF acts upstream of SypG and VpsR to coordinate expression of *syp* and *bcs* genes (Darnell et al., 2008). The SypF-SypG pathway promotes SYP production and cell to cell attachment, while the SypF-VpsR pathway promotes BC production and attachment to surfaces. SypG induces the expression of *binA*, which encodes a GAF-GGDEF-EAL multi-domain protein (Bassis and Visick, 2010). Disruption of *binA* resulted in increased biofilm formation and reduced motility due to increased c-di-GMP levels and increased BC production. BinA acts as a PDE, in which only the EAL domain is significantly conserved. The DGC that promotes BC production in *A. fischeri* is currently unknown. It is postulated that BinA coordinates the temporal increase in SYP production with a decrease in BC production to down-regulate BC biosynthesis in the initial stages of the colonization process (Bassis and Visick, 2010).

The outer membrane protein, OmpU is required for effective colonization and attachment to host tissues (Aeckersberg et al., 2001). A gene required for cysteine metabolism, *cysK*, is required for SYP production, though disruption of *cysK* does not alter the activation of *syp*-encoded proteins (Singh et al., 2015). The coordinated biosynthesis of SYP and BC is a unique association mechanism that illustrates the diversity involved in host colonization by BC producers.

Bacterial cellulose production by *A. fischeri* can be induced by arabinose (Visick et al., 2013). Though it is not found in animal tissue to any significant extent, arabinose is plentiful in seaweed (Rao and Ramana, 1991), suggesting *A. fischeri* may respond to arabinose as part of a plant–bacteria interaction. Seaweeds produce various antimicrobial compounds (Steinberg et al., 1997), so *A. fischeri* BC-containing biofilms would be protected from chemical perils (see review: Davies, 2003). The plant–bacteria interactions of *A. fischeri* have yet to be investigated.

## Fungal–Bacteria Interactions of *S. enterica* Serovars

Another inter-domain interaction is that between *S. enterica* and the phytopathogenic fungus, *Aspergillus niger*. Brandl et al. (2011) demonstrated that numerous *S. enterica* serovars attached to *A. niger* hyphae through a BC–chitin interaction. In their study, a BC-deficient mutant was unable attach to *A. niger*, while re-introduction of BC synthesis genes restored the wild type attachment phenotype. Bacterial attachment to chitin, the main component of the fungal cell wall, occurred strongly with *S. enterica* serovars, and weakly with pathogenic *E. coli* O157:H7, which both produce BC. However, attachment to chitin was not observed for two non-BC producing *Pseudomonas* strains. Curli was not required for attachment and biofilm development on hyphae, but was neccesary for its long-term stability.

Colonization of *A. niger* hyphae by *S. enterica* is a mutualistic interaction. Balbontin et al. (2014) demonstrated that bacterial growth is enhanced upon initiation of the interaction, and that the BC biofilm can protect the fungus from the antifungal agent, cycloheximide. The *S. enterica*–*A. niger* interaction does not negatively affect either participant; co-colonization inhibited the invasion of maize by *S. enterica*. When both *S. enterica* and *A. niger* colonized the roots of *Zea mays*, the negative effect on plant growth was greater than when roots were colonized by either microbe alone.

These studies demonstrate that BC plays a critical role in the attachment of bacteria to chitin within the fungal cell wall, and facilitates tripartite interactions between the bacterium, the fungus, and the host plant. These tripartite associations may be a ubiquitous mechanism that occurs in the rhizosphere, and further emphasizes the important role that BC plays in environmental interactions.

## Concluding Remarks

An important role of BC in environmental biofilms is to facilitate inter-domain interactions, such as those observed between bacteria and their plant, animal or fungal hosts. Biofilms allow bacteria to persist in association with their preferred host or substrate, providing protection against adverse conditions. Colonization of more advanced organisms allows bacteria to take advantage of host-derived nutrients through either symbiotic or pathogenic associations such as on the roots and fruits of plants, or the gut of insects and humans. The ecological diversity of BC-producing organisms underscores the importance of BC as a mediator of environmental interaction. Future studies should be geared toward further elucidating BC-mediated environmental interactions to provide novel insights into the mechanisms that control BC biosynthesis.

## Author Contributions

The authors RA, AV, and JS contributed equally to the preparation of this manuscript.

## Conflict of Interest Statement

The authors declare that the research was conducted in the absence of any commercial or financial relationships that could be construed as a potential conflict of interest.
